# Genome-wide identification and characterization of *ALOG* domain genes in *Rosa*

**DOI:** 10.3389/fpls.2025.1690365

**Published:** 2025-11-20

**Authors:** Feng Chen, Bo Lv, Jiaqi Guo, Jurong Song, Cong Guo, Jie Yang, Jianguo Lin, Yuanyuan Yang, Fayun Xiang

**Affiliations:** 1 Industrial Crops Institute, Hubei Academy of Agricultural Sciences, Wuhan, Hubei, China; 2 Hubei University of Technology/Hubei Key Laboratory of Industrial Microbiology, Wuhan, Hubei, China

**Keywords:** rose, ALOG domain gene, phylogenetic relationships, bioinformatics characteristics, expression patterns, gene function

## Abstract

**Introduction:**

ALOG genes encode transcription factors that control essential growth and developmental processes in various plant species. The ALOG protein domain, which is highly conserved among land plants, exhibits distinct evolutionary patterns in different plant lineages, suggesting its importance in plant adaptation and evolution. *Rosa* (roses), a genus of flowering plants with significant horticultural value, exhibits key traits such as floral organ differentiation and inflorescence architecture diversification. Emerging evidence suggests that ALOG genes not only modulate organogenesis but may also drive evolutionary innovations in floral organ morphology and inflorescence complexity.

**Methods:**

We systematically identified ALOG genes in four *Rosa genomes* (*R. chinensis*, *R. multiflora*, *R. rugosa*, and *R. wichurana*), reconstructed their phylogenetic relationships, and cloned ALOG homologs from *R. chinensis*.

**Results:**

Through integrated bioinformatic analyses including chromosomal localization, protein motif characterization, promoter cis-acting element annotation, and spatiotemporal expression profiling, we provide a comprehensive overview of ALOG gene distribution, structure, and expression patterns in *Rosa*.

**Discussion:**

Our findings provide insights into the potential involvement of *Rosa* ALOG genes in organogenesis and inflorescence patterning, highlighting their possible roles in the evolution of floral morphology and inflorescence complexity.

## 1 Introduction

Roses (*Rosa* spp., Rosaceae), as pivotal species in ornamental horticulture and economic botany, have undergone complex reticulate hybridization events among *Rosa* lineages (Cui et al., 2022). These evolutionary processes have given rise to modern cultivars that are celebrated for their exceptional floral diversity, characterized by vivid pigmentation patterns and extended flowering phenology (Smulders et al., 2019). In recent years, significant progress has been made in elucidating the molecular mechanisms governing rose flower development, photoperiodic regulation of blooming, and postharvest physiology (Jing et al., 2023; Lu et al., 2024). The release of multiple *Rosa* genome assemblies has provided a solid foundation for functional genomic studies, facilitating deeper insights into the genetic mechanisms underlying key traits in roses (Hibrand Saint-Oyant et al., 2018; Liang et al., 2025).

Over the past three decades, researchers have identified a conserved protein family across both dicotyledonous and monocotyledonous plants, initially classified as DUF640 within the Pfam database (Dong et al., 2014; Takanashi et al., 2022; Upadhyaya et al., 2024). This family was subsequently designated the ALOG (*Arabidopsis* Light-dependent Short Hypocotyls 1 and *Oryza* Long Sterile Lemma) family, named after *LSH1* in *Arabidopsis thaliana* (Lee et al., 2020) and *G1* in *Oryza sativa* (Yoshida et al., 2009). ALOG/DUF640 proteins are classified as land plant-specific transcription factors (TFs) based on their characteristics including sequence-specific DNA binding, transcriptional regulatory activity, nuclear localization, and the ability to form homodimers (Huang et al., 2024; Iyer and Aravind, 2012). Extensive studies have characterized *ALOG* genes across the plant kingdom, spanning evolutionarily divergent lineages from bryophytes (Hata et al., 2024; Naramoto et al., 2020; Xiao et al., 2019) to monocotyledonous (Jiang et al., 2024) and eudicotyledonous species (Baranov et al., 2024; Lin et al., 2013; Zirkel et al., 2023). *ALOG* genes are involved in crucial processes of plant growth and developmental processes, including seedling photomorphogenesis, vegetative and reproductive growth, and abiotic stress responses (Liu et al., 2024; Upadhyaya et al., 2024; Vo Phan et al., 2023). *AtLSH1* is the founding member of this gene family and was first characterized in *A. thaliana* (Zhao et al., 2004). The *lsh1-D* mutant exhibited light-dependent dominance, mediating photomorphogenic regulation during seedling development. This mutant exhibited hypersensitivity to sustained far-red, blue, and red light spectra, manifesting significantly reduced hypocotyl elongation compared to wild-type counterparts. Rice *TAWAWA1* (*TAW1*) regulates inflorescence architecture by maintaining meristematic indeterminacy, which sustains inflorescence meristem activity while delaying the transition to spikelet meristem identity, thereby modulating panicle branching complexity (Beretta et al., 2023; Yoshida et al., 2013). Additional *ALOG* members in rice play critical roles in floral organogenesis, where loss-of-function mutations induce homeotic floral abnormalities that directly compromise grain yield (Ma et al., 2013; Peng et al., 2017; Sato et al., 2014). *AtLSH8* is a novel positive regulator of the ABA signaling pathway. When its function is lost, *Arabidopsis* becomes insensitive to ABA treatment, primarily because of altered expression patterns of ABA-responsive proteins in the mutant (Zou et al., 2021).

As research on *ALOG* genes has advanced, their evolutionary trajectories and roles in plant speciation have become key topics of investigation. When *OsG1* is functionally impaired, the morphology of the sterile lemma in rice undergoes significant changes and develops into a structure resembling the lemma in form and size. This phenotype, characterized by an enlarged sterile lemma equivalent to the lemma, naturally occurs in *Oryza grandiglumis*, and the *G1* gene in *O. grandiglumis* exhibits distinct mutations (Yoshida et al., 2009). The tomato *TERMINATING FLOWER* (*TMF*) gene governs inflorescence determinacy through temporal suppression of floral meristem identity genes. The *tmf* mutant developed solitary-flowered primary inflorescences owing to the precocious activation of *ANANTHA* (*AN*) and *SEPALLATA3* (*SEP3*) in vegetative meristems, coupled with a delayed flowering transition. Crucially, experimental modulation of *AN* expression timing recapitulates diverse inflorescence architectures, suggesting that this regulatory mechanism represents an evolutionary substrate for inflorescence diversification within the Solanaceae (MacAlister et al., 2012). Constitutive expression of *AtLSH3* leads to abnormal fusion of the anthers and petals, suggesting that *AtLSH3* is a potential modulator of sympetaly, the developmental program underlying fused corolla formation (Cho and Zambryski, 2011). During evolution, to protect flowers from various abiotic stresses, such as rain-induced damage to pollen and nectar, *Torenia fournieri* has developed a specialized structure known as the corolla neck (Nachev et al., 2017). *TfALOG3* has been demonstrated to play a role in the evolutionary development of the corolla neck, as evidenced by its specific expression in the corolla neck and the absence of this structure in the *tfalog3* mutant (Xiao et al., 2019). In legumes, which have evolved specialized root nodules, ALOG-domain genes have been shown to play key developmental roles that are not only closely associated with nodulation processes (Ahmad et al., 2024), but also contribute to the establishment of zygomorphic floral symmetry, a defining feature of legume floral architecture (He et al., 2020; Lee et al., 2024; Lei et al., 2019).

Floral morphogenesis and inflorescence patterning are key developmental processes in *Rosa*; however, the molecular regulators underlying these traits remain largely unexplored. Although *ALOG* genes are known to regulate reproductive development in diverse plant lineages, their roles in the family Rosaceae have not yet been characterized. We performed a genome-wide identification of *ALOG* homologs in four *Rosa* species and cloned the full-length coding sequences (CDSs) of *ALOG* genes from *R. chinensis* ‘Old Blush’, referred to as *RcLSH* genes. We further conducted phylogenetic analysis, gene structure analysis, and motif characterization, as well as investigated the conserved domain organization. Spatiotemporal expression profiling using qPCR revealed different patterns of *RcLSH* expression across tissues and developmental stages, offering insights into its potential functions in rose organogenesis and inflorescence development.

## 2 Materials and methods

### 2.1 Plant materials and conditions

Seedlings of *R. chinensis* ‘Old Blush’ were generously provided by Professor Guogui Ning from Huazhong Agricultural University, China. The plants were grown in a greenhouse under natural photoperiod conditions, with a relative humidity of 70%–85% and a temperature range of 20–28°C.

Plant samples were collected from various tissues and developmental stages of *R. chinensis* ‘Old Blush’, including vegetative organs (prickles, leaves, roots, and stems), reproductive organs (0.5cm floral buds, floral receptacle, pedicel, sepals, petals, stamens, pistils, and ovaries from newly opened flowers) and shoot apices at four developmental stages (two-leaf, four-leaf, six-leaf, and eight-leaf stages). All samples were immediately frozen in liquid nitrogen and stored at −80°C until use.

### 2.2 Identification of *ALOG* Genes in *Rosa*

Four publicly available *Rosa* genomes were used to identify *ALOG* genes, including *R. chinensis*, *R. wichurana*, *R. multiflora*, and *R. rugosa*. Candidate *ALOG* genes were identified using both BLASTP and Hidden Markov Model (HMM) searches. The BLASTP search was conducted in the Rosaceae genome database (Jung et al., 2019), with known ALOG proteins from *A. thaliana* and *O. sativa* as queries. In parallel, the HMM profile of the DUF640 domain (PF04852) obtained from the Pfam database was used to perform an HMMER search against annotated *Rosa* protein sequences. The combined results were further verified to ensure all candidates contained the conserved ALOG domain, and redundant or incomplete sequences were manually curated and removed.

### 2.3 Cloning of the *ALOG* gene in *R. chinensis* ‘Old Blush’

Total RNA was extracted using the RNA extraction kit (Aidlab, China). cDNA was synthesized using PrimeScript RT reagent kit with genomic DNA Eraser (Takara, Japan) from 2 μg of total RNA per sample. To validate the identified genes, gene-specific primers were designed based on the predicted CDS regions. The same primer pairs were used to amplify both the full-length CDS from cDNA and the corresponding genomic sequences (including introns) from genomic DNA. PCR amplification was performed with 2× PrimeSTAR Max Premix (Takara, Japan), a high-fidelity enzyme, and 2 μl of diluted mixture cDNA (1:20) in the PCR system. The PCR products were purified using a DNA Gel Extraction Kit (Aidlab Biotech, China) and cloned into the pMD18-T vector (Takara, Japan). The plasmids were then transformed into *E. coli* Top 10 via heat shock. For each gene clone, at least three positive clones were selected for sequencing (TSINGKE Biotechnology, China), and sequences with consistent results from two single clones were identified as the accurate gene sequence.

### 2.4 Multiple sequence alignment and phylogenetic analysis

Multiple sequence alignment and phylogenetic analyses were performed using MEGA 6.0 (Tamura et al., 2013). A total of 81 ALOG proteins from nine representative species were included: 8 from *R. chinensis*, 7 from *Fragaria vesca*, 10 from *A. thaliana* (Zhao et al., 2004), 10 from *O. sativa* (Yoshida et al., 2009), 4 from *Physcomitrium patens*, 11 from *Petunia hybrida* (Chen et al., 2019), 12 from *S. lycopersicum* (Turchetto et al., 2023), 7 from *T. fournieri* (Xiao et al., 2018), and 12 from *Lotus japonicus* (Lei et al., 2019). Gene nomenclature was obtained from published literature, except for *F. vesca*.

Amino acid sequences were aligned using the MUSCLE program in MEGA 6.0. Based on the conserved ALOG domain, a maximum likelihood (ML) tree was constructed using the JTT+G+I model with 1,000 bootstrap replicates. Additionally, a neighbor-joining (NJ) tree of the *LSH* genes in the four *Rosa* species was built using MEGA 6.0, with sequences aligned by MUSCLE, evolutionary distances calculated using the Poisson model, and branch support evaluated with 1,000 bootstrap replicates.

### 2.5 Bioinformatics analysis of ALOG genes in *R. chinensis*

We conducted analyses of the biological characteristics of *ALOG* genes in *R. chinensis*, including gene structure, motif analysis, promoter cis-acting element analysis, and chromosomal localization. The exon-intron structure of *ALOG* genes in the four *Rosa* species was analyzed using the Gene Structure Display Server (Hu et al., 2015). Motifs in RcLSH proteins were predicted using MEME Suite 4.12.0 with the following parameters: Minimum motif width = 6, Maximum motif width = 50, Maximum number of motifs = 20 (Bailey et al., 2009). The promoter regions, defined as 2,000 bp upstream of the start codon of *ALOG* genes in *R. chinensis*, were analyzed for cis-acting regulatory elements using the PlantCARE database (Lescot et al., 2002). Identified elements were categorized and visualized using TBtools (Chen et al., 2020). Chromosomal localization was performed using the MG2C online platform (Chao et al., 2021).

### 2.6 Spatiotemporal expression patterns of *ALOG* genes in *R. chinensis*

Quantitative real-time PCR (qRT-PCR) was performed using SYBR Premix Ex Taq (Takara, Japan) on a BIO-RAD CFX384 Touch Real-Time PCR Detection System (Bio-Rad Laboratories, USA). The reaction was conducted in a 10 μL volume with 1 μL of 20× diluted cDNA template, 5 μL of 2× SYBR Green Master Mix, 0.2 μL of forward and reverse primers (10 μmol/μL each), and sterile water to adjust the final volume. The transcription level of *RcUBC* was used as the reference gene (Song et al., 2023). Each reaction was performed in three biological replicates, and the results were calculated as mean ± standard deviation. The 2^−ΔΔCT^ method (Livak and Schmittgen, 2001) was used for data analysis.

## 3 Results

### 3.1 Genome-wide identification of *ALOG* genes in *Rosa*

Using ALOG protein sequences from *A. thaliana* and *O. sativa* as queries, we conducted a genome-wide identification of *ALOG* homologs across four *Rosa* species through BLASTP and HMMER searches. Comparative genomic analysis revealed species-specific expansions (**Supplementary File 1**): eight *ALOG* genes were identified in *R. chinensis*, *R. rugosa* and *R. multiflora* (*RcLSHs*, *RrLSHs* and *RmuLSHs*), and ten in *R. wichurana* (*RwLSHs*). Notably, sequence alignment indicated 100% identity between Rw0G017960 and Rw0G022240 in *R. wichurana*; thus, only Rw0G017960 was retained for subsequent analyses to avoid redundancy. All identified genes were systematically numbered based on their orthology with *Arabidopsis ALOG* members (**Supplementary File 2**).

To validate the predicted coding sequences (CDS) and exon-intron architectures of *Rosa ALOG* genes, we designed gene-specific primers (**Supplementary File 3**) and amplified both the CDS and genomic DNA (gDNA) from *R. chinensis* ‘Old Blush’. All the predicted *ALOG* genes in *R. chinensis* were successfully amplified and validated by sequencing (**Table 1**).

**Table 1:** Gene features of *ALOG* genes in *R. chinensis* ‘Old Blush’.

Gene name	NCBI accession number	Gene length (bp)	ORF length (bp)	Protein length (aa)
*RcLSH1*	PV779459	636	636	211
*RcLSH2*	PV779460	744	744	247
*RcLSH3*	PV779461	636	636	211
*RcLSH4*	PV779462	603	603	200
*RcLSH5*	PV779463	693	693	230
*RcLSH7*	PV779464	582	582	193
*RcLSH10a*	PV779465	609	609	202
*RcLSH10c*	PV779466	558	558	185

### 3.2 Sequence characteristics of *Rosa ALOG* genes

**Figure 1:**
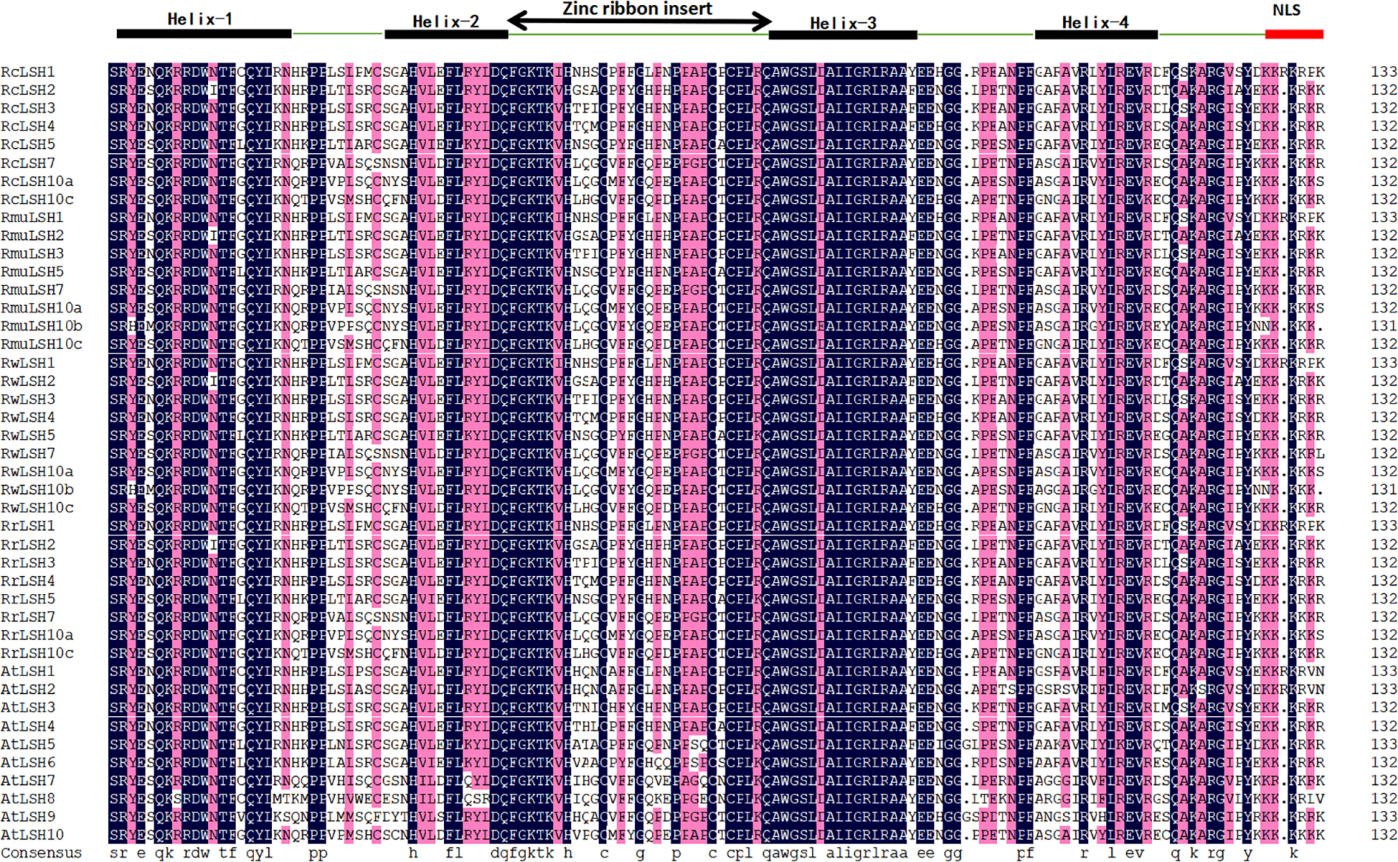
Conserved ALOG domain structure in four *Rosa* species. The ALOG domain consists of four α-helices, a zinc ribbon insert, and a C-terminal nuclear localization signal (NLS). Residues in black are fully conserved, while purple indicates >75% sequence similarity. Species abbreviations: Rc, *R. chinensis*; Rmu, *R. multiflora*; Rw, *R. wichurana*; Rr, *R. rugosa*; At, *A. thaliana*.

**Figure 2:**
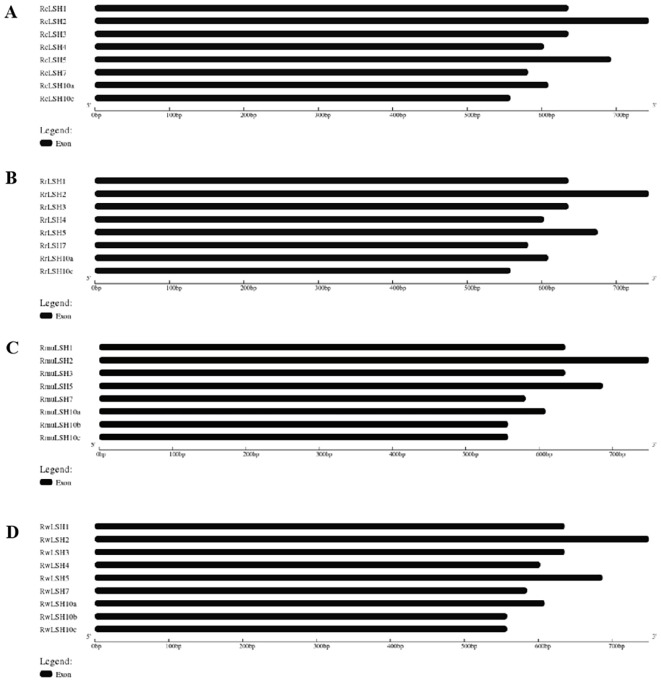
Gene structures of *ALOG* genes in four *Rosa* species. All genes in *R. chinensis***(A)**, *R. multiflora***(B)**, *R. wichurana***(C)** and *R. rugosa***(D)** were intronless. Species abbreviations: Rc, *R. chinensis*; Rmu, *R. multiflora*; Rw, *R. wichurana*; Rr, *R. rugosa*.

All *ALOG* genes identified in the four *Rosa* genomes contained the conserved ALOG domain (**Figure 1**) but lacked introns, as demonstrated by exon–intron structure analysis (**Figure 2**). Comparative sequence analysis revealed a remarkable consistency in gene length among species. The CDS length of the *ALOG* genes ranged from 558 to 750 bp across the four *Rosa* species, corresponding to proteins of 185–249 amino acids. Notably, all species shared a minimum CDS length of 558 bp (185 aa). The maximum CDS length reached 744 bp (247 aa) in *R. chinensis* and *R. rugosa*, while 750 bp (249 aa) in *R. wichurana* and *R. multiflora* (**Supplementary File 1**).

### 3.3 Phylogenetic analysis of RcLSH proteins

**Figure 3:**
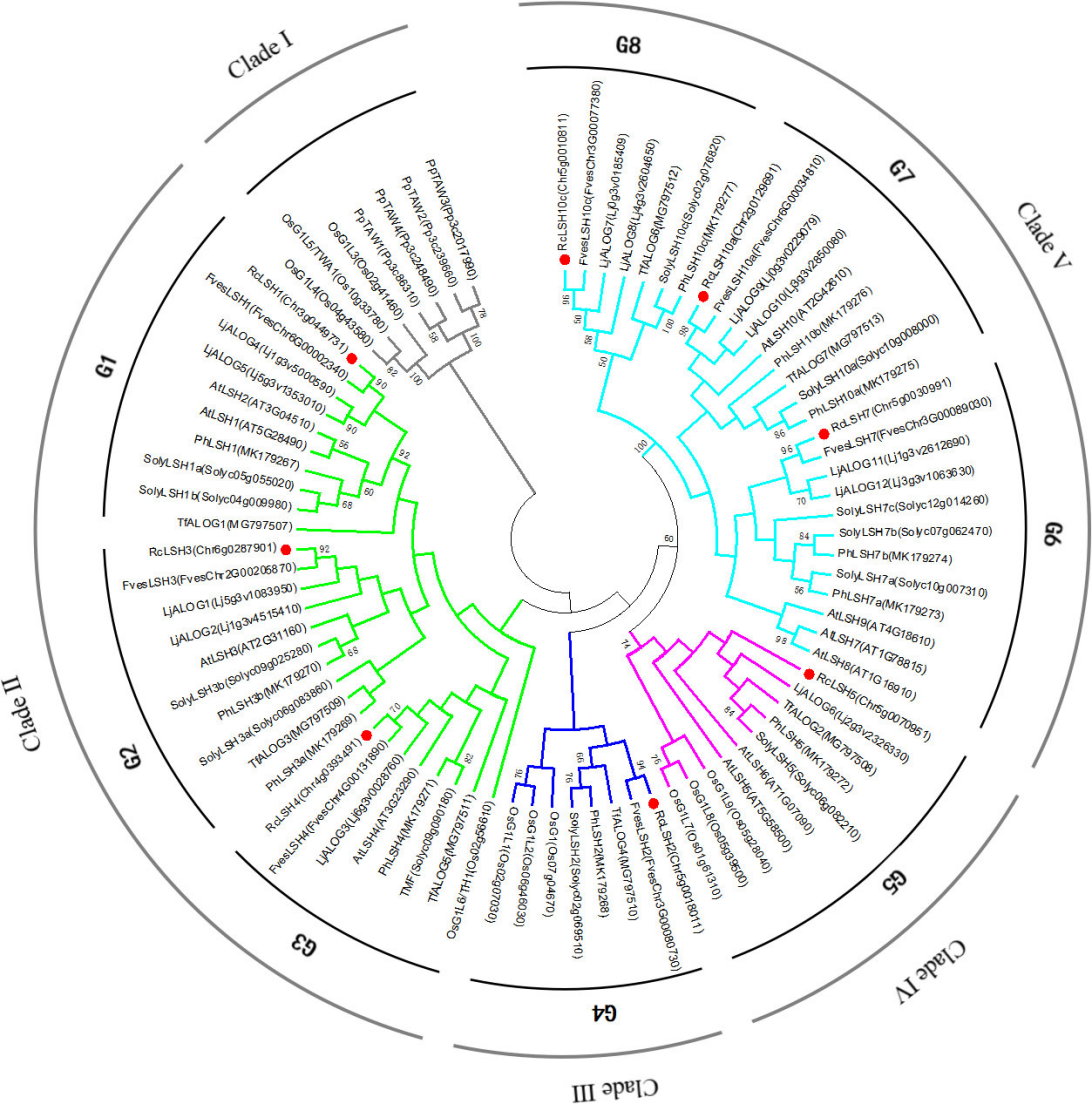
Phylogenetic analysis of ALOG proteins from nine representative species. 81 ALOG proteins from nine representative species were aligned using MUSCLE in MEGA 6.0. A maximum likelihood (ML) tree was constructed with the JTT+G+I model and 1,000 bootstrap replicates. Branch colors indicate distinct evolutionary clades. ALOG proteins are classified into five major clades and eight subgroups. Species abbreviations: Pp, *P. patens*; Rc, *R. chinensis*; At, *A. thaliana*; Soly, *S. lycopersicum*; Ph, *P. hybrida*; Tf, *T. fournieri*; Lj, *L. japonicus*; Fves, *F. vesca*; Os, *O. sativa*.

To resolve the evolutionary trajectory of ALOG proteins, we constructed a phylogenetic tree using sequences from nine strategically selected species (**Supplementary File 4**), including *P. patens* (as a basal bryophyte and the root of evolution), dicotyledons (*R. chinensis*, *A. thaliana*, *S. lycopersicum*, *P. hybrida*, *T. fournieri*, *L. japonicus*, and *F. vesca)*, and a monocot (*O. sativa*). The inclusion of these taxa, representing distinct evolutionary lineages with varied levels of *ALOG* gene characterization enabled robust functional inference of *Rosa ALOG* homologs. Phylogenetic reconstruction of ALOG proteins from the selected species resolved the five well-supported clades (**Figure 3**). Clade I, which represented the basal group of the phylogenetic tree, contained ALOG proteins from *P. patens* and *O. sativa*. Clade II (AtLSH1/2/3/4-like) included representatives of all nine species, suggesting ancestral conservation. Clades III and IV (AtLSH5/6-like) also included ALOG proteins from both monocots and dicots, but with relatively fewer members, which may indicate the loss of this gene subfamily in certain dicot species. In contrast, Clade V (AtLSH7/8/9/10-like), a type of dicot-specific radiation, consisted exclusively of ALOG proteins from dicots. To further elucidate the evolutionary relationships among *R. chinensis* ALOG proteins, we subdivided the *R. chinensis* ALOG proteins into eight distinct subgroups (G1–G8), each containing a single ALOG protein from *R. chinensis*. Specifically, G4 and G8 lack *Arabidopsis* ALOG orthologs, implying that G4 and G8 may represent a lineage-specific specialization of ALOG proteins.

### 3.4 Genomic dynamics and evolutionary trajectories of *ALOG* genes in *Rosa*

**Figure 4:**
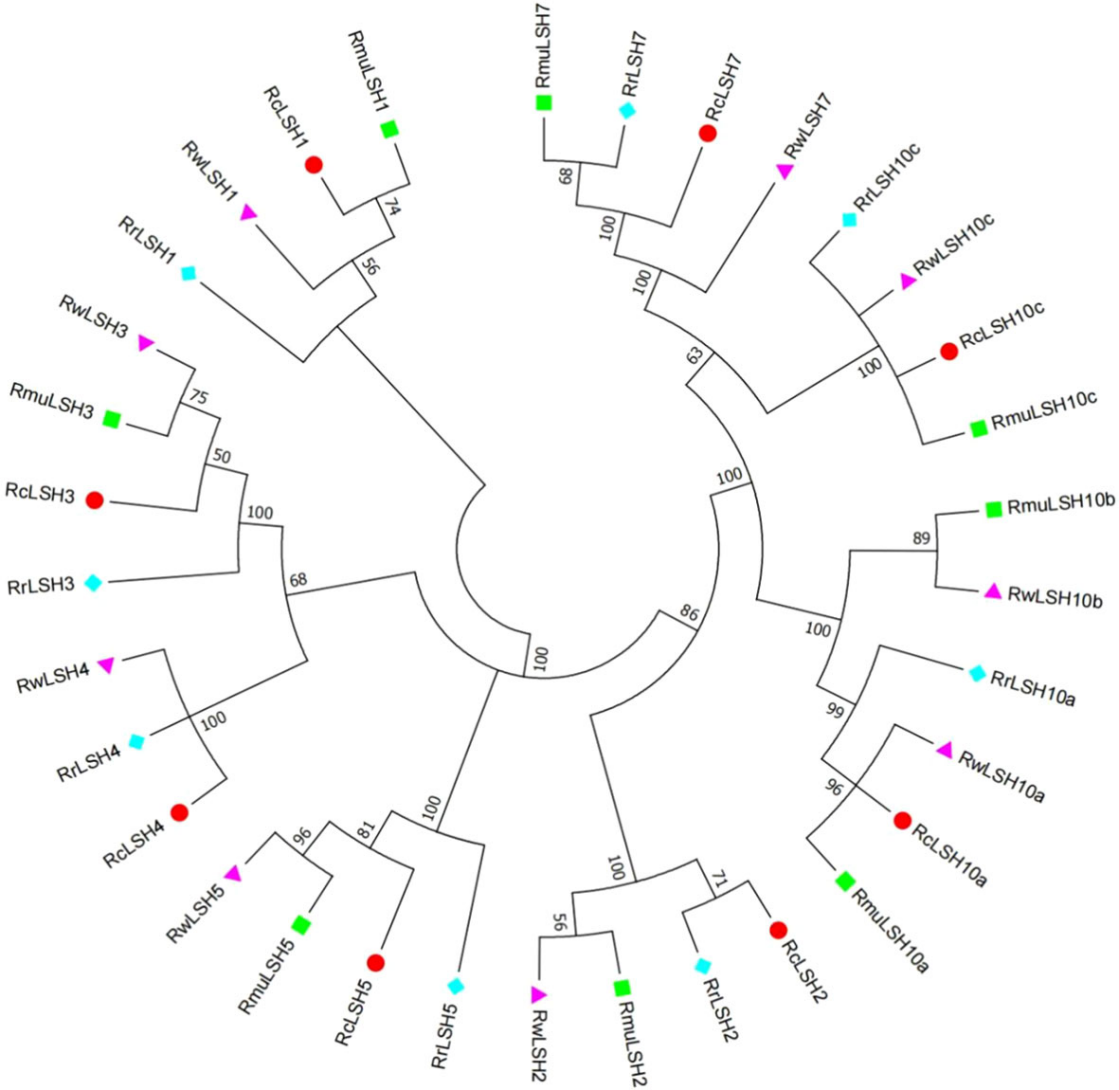
Phylogenetic relationships of *ALOG* genes among four *Rosa* species. Full-length CDS sequences of *ALOG* genes from four *Rosa* species were aligned using MUSCLE. A neighbor-joining (NJ) tree was generated in MEGA 6.0 with 1,000 bootstrap replicates. Species abbreviations: Rc, *R. chinensis*; Rmu, *R. multiflora*; Rw, *R. wichurana*; Rr, *R. rugosa*.

Genome-wide identification revealed the dynamic evolutionary patterns of *ALOG* genes across *Rosa* species, marked by interspecific variations in gene copy numbers and chromosomal distribution patterns (**Supplementary File 1**). To resolve the lineage-specific diversification mechanisms, we reconstructed a phylogenetic tree using CDS sequences from the four *Rosa* genomes. This genus-wide phylogeny demonstrates recurrent gene loss and divergence events that shape the extant ALOG repertoires (**Figure 4**). Notably, *LSH4* homologs were exclusively absent in *R. multiflora*. *LSH10b* underwent functional diversification in *R. multiflora* and *R. wichuriana*, yielding distinct paralogs that were absent in *R. chinensis* and *R. rugosa*.

### 3.5 Integrative analysis of chromosomal distribution, protein motifs, and regulatory elements

**Figure 5:**
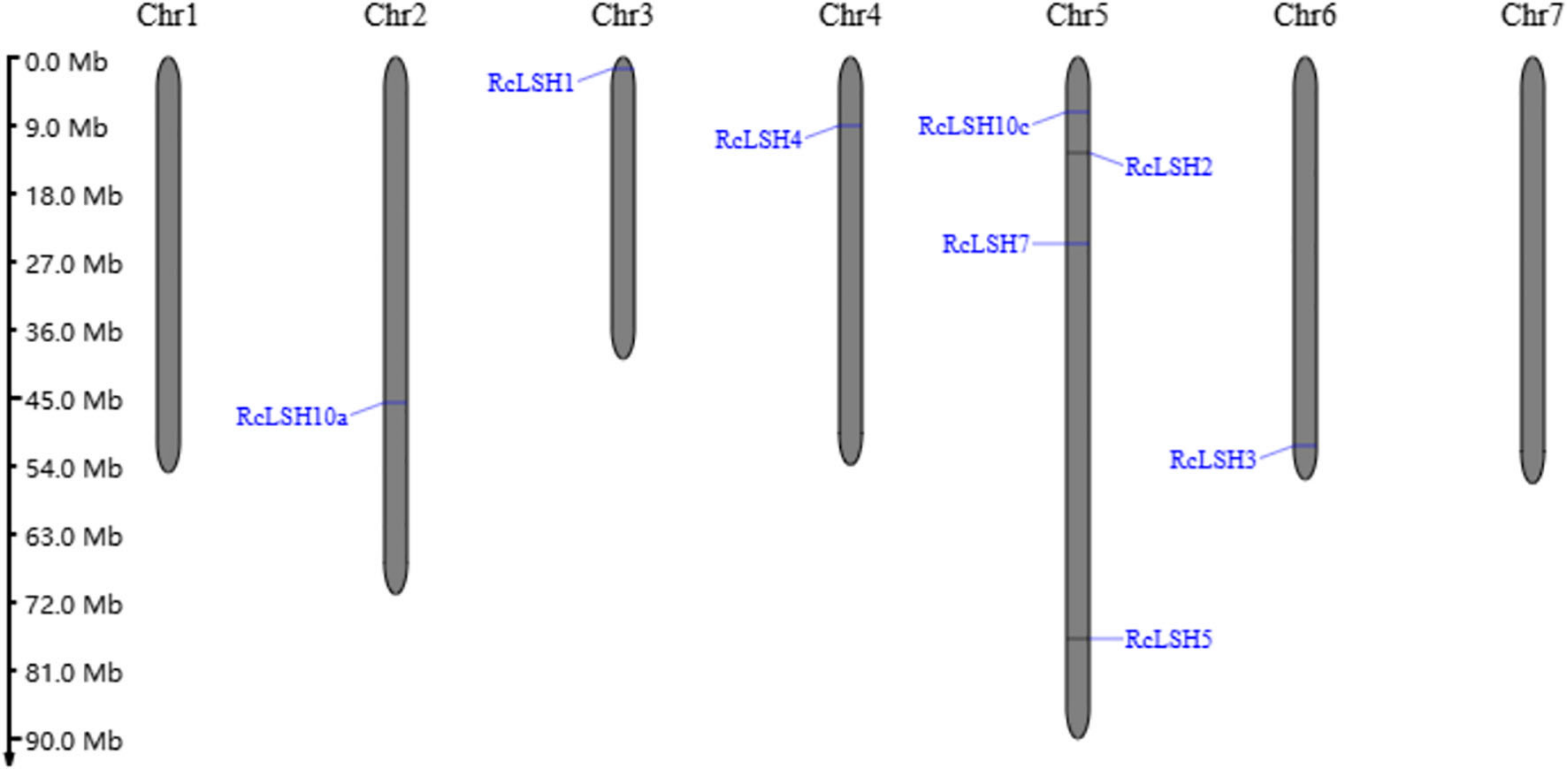
Chromosomal distribution of *ALOG* genes in *R. chinensis*. Chromosomal positions were extracted from the *R. chinensis* ‘Old Blush’ reference genome and mapped to chromosomes.

**Figure 6:**
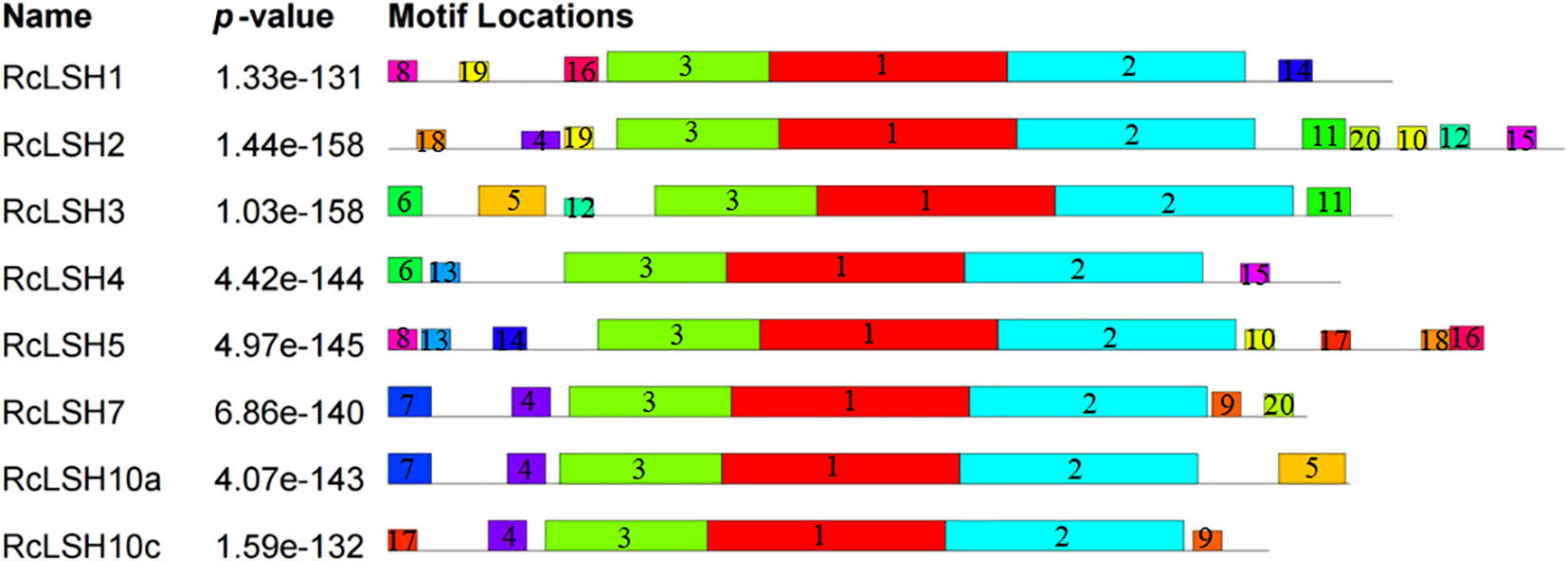
Conserved motif composition of ALOG proteins in *R. chinensis*. Different colored boxes represent distinct predicted motifs, and the Arabic numerals above the motifs indicate their corresponding motif numbers. Motif analysis was performed in MEME.

**Figure 7:**
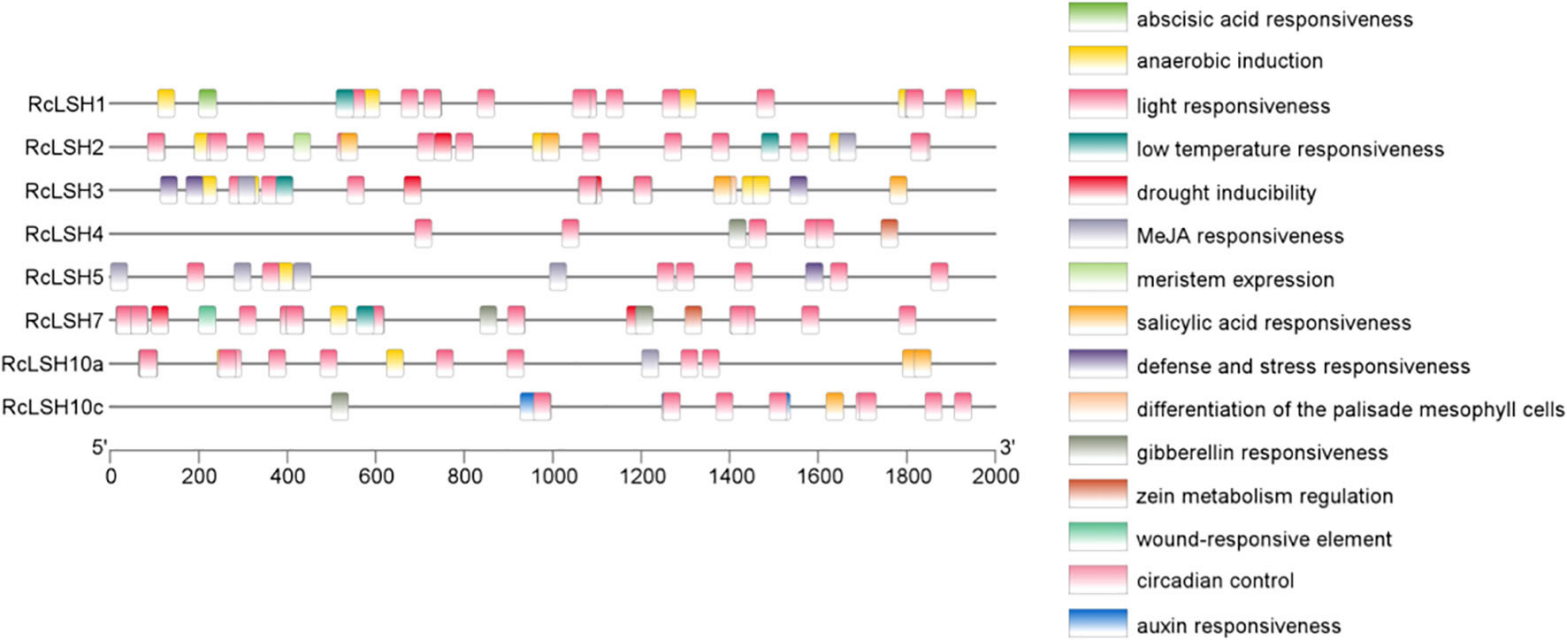
Cis-regulatory element analysis of *ALOG* gene promoters in *R. chinensis*. Promoter sequences (2000 bp upstream of the ATG) were extracted from the *R. chinensis* genome and analyzed with PlantCARE. Cis-elements were grouped by functional category and displayed with TBtools.

Chromosomal mapping revealed a non-random distribution of *RcLSH* genes across five of the seven chromosomes in *R. chinensis* (**Figure 5**). Notably, chromosome 5 harbored a gene cluster containing four *RcLSH* paralogs (*RcLSH2*, *RcLSH5*, *RcLSH7*, and *RcLSH10c*), suggesting potential tandem duplication events. Conserved motif analysis identified three functionally critical motifs within the ALOG domain (**Figure 6**; **Supplementary File 5**), that were predicted to mediate DNA-binding and protein interaction activities. Intergroup motif distribution heterogeneity among the eight RcLSH proteins aligned with their phylogenetic classification, reinforcing structure–function conservation within evolutionary subgroups. Profiling of promoter cis-elements identified 15 distinct regulatory elements in the RcLSH promoters (**Figure 7**; **Supplementary File 6**; **Supplementary Table 1**). Light-responsive elements were predominant, whereas hormone- and stress-responsive elements were also present.

### 3.6 Spatiotemporal expression pattern of *ALOG* genes in *R. chinensis*

Gene functionality is closely linked to spatiotemporal expression dynamics. To decipher the biological roles of *ALOG* genes in *R. chinensis*, we conducted qRT-PCR analysis across 12 tissue types including prickles, leaves, roots, stems, floral buds, receptacles, pedicels, sepals, petals, stamens, pistils, and samples from different developmental stages shoot apices.

**Figure 8:**
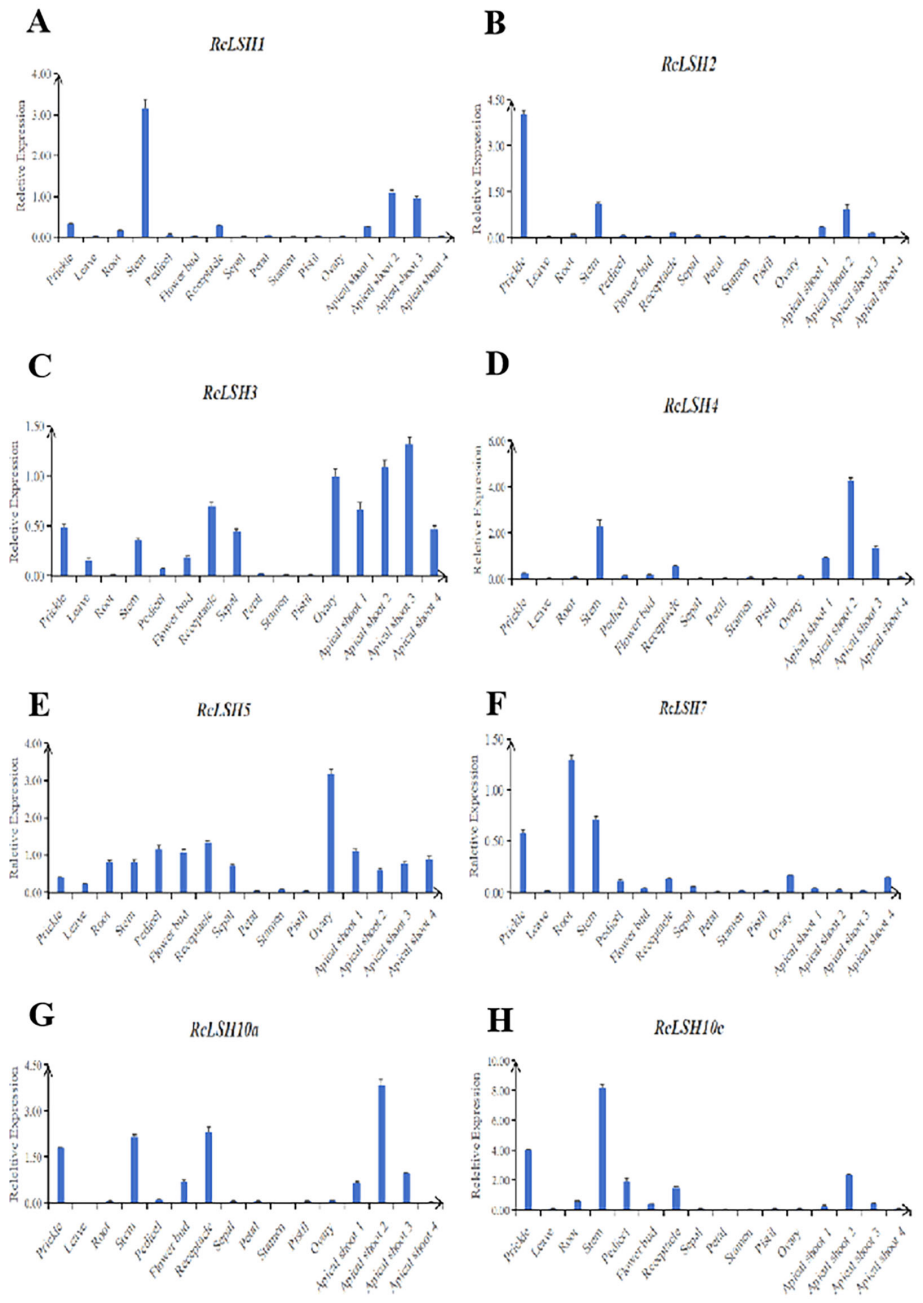
Spatiotemporal expression patterns of *ALOG* genes in *R. chinensis*. qRT-PCR was performed using SYBR Premix Ex Taq on a BIO-RAD CFX384 system. *RcUBC* was used as the reference gene, and expression levels were calculated with the 2^−ΔΔCT^ method. Samples included vegetative tissues (prickles, leaves, roots, stems), reproductive organs (buds, sepals, petals, stamens, pistils, ovaries), floral receptacles, pedicels, and shoot apices at four developmental stages. Expression values were averaged across three replicates. *RcLSH1***(A)**, *RcLSH2***(B)**, *RcLSH3***(C)**, *RcLSH4***(D)**, *RcLSH5***(E)**, *RcLSH7***(F)**, *RcLSH10a***(G)** and *RcLSH10c***(H)**.

The results revealed distinct expression patterns among different *ALOG* genes in *R. chinensis* (**Figure 8**; **Supplementary File 7**). *RcLSH1* is highly expressed in stems and was also significantly expressed in early stage shoot apices. *RcLSH2* was predominantly expressed in prickles, followed by stems, and was notably expressed in early stage shoot apices. *RcLSH3* displayed the highest expression in the shoot apices, with transcript levels gradually decreasing as the shoot apex developed. It is also expressed in various floral and vegetative organs, with the highest expression observed in the ovary. *RcLSH4* exhibited the highest expression in the stem segments, followed by the prickles; however its expression was significantly up-regulated only during the mid-stage of vegetative growth in shoot apices. *RcLSH5* is broadly expressed across different tissues and developmental stages, except in the petals, stamens, and pistils. Notably, the highest expression levels were observed in the ovaries. *RcLSH7* was predominantly expressed in roots, stems, and prickles, whereas its expression in other tissues was relatively low. *RcLSH10a* and *RcLSH10c* exhibited similar expression patterns, characterized by low expression in floral organs but significant expression in stems, prickles, and the second stage of shoot apex development. These findings suggest that *R. chinensis ALOG* genes exhibit tissue- and stage-specific expression, indicating their potential roles in organ differentiation, shoot apical meristem development, and reproductive organ formation.

## 4 Discussion

In this study, we systematically identified ALOG family members in four *Rosa* species and constructed a phylogenetic tree using *ALOG* genes from different species. We cloned *R. chinensis* genomic and CDS sequences and analyzed their gene structure, chromosomal localization, motif composition, promoter cis-elements, and spatiotemporal expression patterns. These analyses provide important insights into the evolution and potential roles of *Rosa ALOG* genes in growth and development. However, gene function studies typically rely on stable genetic transformation systems (such as overexpression, downregulation, or even gene knockout) that are not yet well established in *Rosa* and require lengthy research periods. Therefore, our study not only offers new perspectives on the systematic analysis of *ALOG* genes in *Rosa*, but also lays the foundation for identifying genes involved in the development of important traits in *Rosa*, especially those related to floral and specialized organ development, providing a reference for future functional studies and validation.

Compared with other dicotyledonous plants, the number of *ALOG* genes in the *Rosa* genus is moderate. For instance, strawberries, another member of the Rosaceae family, contain seven *ALOG* genes, whereas both *A. thaliana* and *O. sativa* each harbor ten *ALOG* genes (Iyer and Aravind, 2012; Zhao et al., 2004; Yoshida et al., 2009). Similar to *ALOG* genes in other species, *Rosa ALOG* genes possess a highly conserved ALOG domain characterized by four α-helices, a zinc finger insert, and a nuclear localization signal (Chen et al., 2019). These conserved structural features are essential for their role as transcription factors. Interestingly, all *ALOG* genes identified in *Rosa* lack introns, which is somewhat distinct from other species where most *ALOG* genes are intronless, although a few still contain introns (Chen et al., 2019). This observation suggests that the intronless structure of *ALOG* genes may represent a characteristic evolutionary pattern of this genus. It may also reflect structural conservation and simplification during evolution, potentially contributing to more efficient gene function in plant development. However, further studies are required to elucidate the functional and evolutionary implications of this feature in *Rosa*. In *R. chinensis*, *ALOG* genes were not randomly distributed across all chromosomes; notably, four genes were located on chromosome 5. This clustered arrangement likely reflects tandem duplication events, which may have contributed to the expansion and diversification of *ALOG* gene function within the genus. Such structural conservation and lineage-specific expansion suggest potential subfunctionalization of *ALOG* genes in *Rosa*, which is supported by their diverse expression profiles.

We were particularly interested in exploring the potential biological functions of *ALOG* genes in *Rosa* species, as *ALOG* genes play crucial roles in plant growth and development in other species, especially flower development (Chen et al., 2019; Li et al., 2019; Nan et al., 2018). Given that roses are a well-known ornamental flower, understanding the functions of its *ALOG* genes is of great significance. In this study, we constructed a phylogenetic tree of *ALOG* genes in *R. chinensis*, including species for which *ALOG* genes have been functionally characterized, except for *F. vesca* (strawberry), a representative species within the Rosaceae family, to validate the reliability of our phylogenetic analysis. Because gene expression patterns are closely related to gene function, we further analyzed the spatiotemporal expression profiles of *Rosa ALOG* genes. By integrating the phylogenetic clustering and expression data, we inferred the potential biological roles of these genes in growth and development.

Phylogenetic analysis classified the 81 ALOG proteins from nine different species into five well-defined clades. Clade I formed the basal lineage, exclusively comprising all *P. patens* ALOG proteins alongside three rice homologs. This topology aligns with broader phylogenetic analyses of 458 ALOG proteins across 61 species, which identified ancestral ALOG lineages encompassing bryophytes and selected monocots (Turchetto et al., 2023). Clades II–V exhibited angiosperm-specific diversification with pronounced lineage specialization.

Clade II comprised three subgroups (G1, G2, and G3) corresponding to the *A. thaliana* lineages AtLSH1/2/3/4. G1 contains the *RcLSH1* gene from *R. chinensis*. Within this clade, *AtLSH1* was the first functionally characterized *ALOG* involved in hypocotyl development and is regulated by different light qualities (Zhao et al., 2004). *AtLSH2* is a homolog of *AtLSH1* and exhibits a similar expression pattern. However, the loss of function of both *LSH1* and *LSH2* does not result in a visible phenotype, suggesting the possible involvement of other homologous genes, such as *AtLSH9* in clade G6 (Zhao et al., 2004). The expression patterns and functional characteristics of *AtLSH1* and *AtLSH9* are highly similar, implying a potential functional redundancy within the ALOG gene family (Press and Queitsch, 2017). Given the highly conserved nature of the ALOG domain, this redundancy may be a common feature among ALOG family members. Therefore, the functional characterization of this gene family likely requires the generation of double or multiple mutants. In contrast, *RcLSH1* was significantly expressed in the stems of *Rosa* and was notably expressed in the shoot apices at different developmental stages. This suggests that *RcLSH1* may be involved in stem and shoot apex development, indicating functional divergence from its homolog in *Arabidopsis*. Similar results were observed for *TfALOG1* within the same subgroup, which was highly expressed in flower buds at different stages of *T. fournieri*. Overexpression of *TfALOG1* in *T. fournieri* leads to the flattening of conical cells in the petals, altered petal morphology, elongation of the yellow region in the petal tube, and changes in leaf coloration. These findings indicate that *TfALOG1* plays a critical role in organ development and regulates floral organ formation, which is consistent with its expression patterns (Xiao et al., 2018). *LjALOG4* and *LjALOG5* were highly expressed in the stem and shoot apical meristems (SAM), similar to the expression pattern observed for *RcLSH1* in *Rosa*. Researchers have focused on the potential roles of *LjALOG4/5* in root nodule formation in *L. japonicus*. Although direct evidence is currently lacking, it is highly likely that *LjALOG4/5* are involved in stem, shoot apex, and root nodule development (Lei et al., 2019). Notably, although the expression pattern of *RcLSH1* differed from that of *AtLSH1*, promoter analysis revealed that all *RcLSH* gene promoters contained numerous light-responsive elements, suggesting that light may play a regulatory role in these genes.

In subgroup G2, *AtLSH3* was specifically expressed in the SAM and the lateral organs, predominantly in the boundary regions between these structures. Functional studies have shown that overexpression of *AtLSH3* alters petal number and size and leads to petal–stamen fusion, whereas ablation of *AtLSH3*-expressing cells results in the loss of SAM and lateral organs. The expression patterns and biological functions of *AtLSH3* are largely similar to those of *AtLSH4* (subgroup G4), and both genes have been proposed to function as suppressors of organ differentiation in the boundary domains (Cho and Zambryski, 2011; Rieu et al., 2024; Takeda et al., 2011). Consistently, *RcLSH3* and *RcLSH4* in *R. chinensis* exhibited pronounced expression in the shoot apex, with transcript levels displaying a dynamic pattern: initially low, then markedly elevated, and subsequently declining as the terminal bud progressed. Such temporal variations in expression are likely associated with the transition of the shoot apex from vegetative to reproductive growth, a process that is also well-characterized in tomatoes. In tomato, the *TMF* gene (subgroup G4) plays a crucial role in modulating the inflorescence architecture by repressing the activity of the inflorescence meristem, primarily through the regulation of its downstream gene *AN*.

Furthermore, several *ALOG* genes in tomato, including *TFAM2* (*SolyLSH1a*) in G1, *TFAM3* (*SolyLSH3b*) and *TFAM11* (*SolyLSH3a*) in G2, and *TFAM1* (*SolyLSH2*) in G4 exhibited expression dynamics comparable to *TMF* in the SAM. Both *TFAM1* and *TFAM2* have been demonstrated to interact directly with *TMF* to form heterodimers. These homologous and heterologous interactions among *TMF*, *TFAM1*, and *TFAM2* suggest the existence of ALOG transcriptional complexes that act cooperatively to regulate shoot and early floral meristem development (MacAlister et al., 2012; Xu et al., 2016; Lippman, 2018). Notably, *TFAM3* has been shown to participate in inflorescence development, whereas *TFAM11* does not contribute to this process (Huang et al., 2022). Given these insights, it is reasonable to hypothesize that *ALOG* genes in roses play conserved yet diverse roles in regulating floral transition and inflorescence development. In support of this, we observed that *RcLSH1* (G1), *RcLSH4* (G3), *RcLSH2* (G4), *RcLSH10a* (G7), and *RcLSH10c* (G8) displayed dynamic expression patterns at the shoot apex similar to those reported in tomatoes. Therefore, we propose that these *RcLSHs* are potential regulators of inflorescence architecture in roses, which warrants further functional characterization.

Clade III was characterized by the absence of *A. thaliana* and *L. japonicus* ALOG proteins. This unique lineage, corroborated by independent studies (Chen et al., 2019; Xiao et al., 2018), provides evidence of late duplication events of *ALOG* genes within Brassicaceae and Fabaceae. Within this clade, *RcLSH2* exhibited significant expression not only in the shoot apex but also in the prickles of *R. chinensis.* The prickles of *Rosa* species represent evolutionarily specialized epidermal structures that have emerged as adaptive morphological innovations in response to environmental pressures (Zhang et al., 2024). These structures may share developmental and evolutionary connections with the ALOG-domain genes. The potential role of *RcLSH2* in prickle development warrants functional validation through experimental approaches such as mutant analysis or transgenic studies. Considering the expression pattern of *RcLSH2* and the fact that this subgroup also includes three rice *ALOG* genes—*OsG1*, *OsG1L1* and *OsG1L2*—that are involved in floral organ and inflorescence development (Beretta et al., 2023; Yoshida et al., 2009), we hypothesized that *RcLSH2* participates in the regulation of both inflorescence and prickle development in roses. The putative involvement of *RcLSH2* in prickle formation may represent a possible neofunctionalization event within *Rosa*, whereby *ALOG* genes have been recruited into novel epidermal developmental pathways.

Clade IV contained *RcLSH5* (G5), which was expressed in most tissues, with the highest expression levels in ovules, suggesting a potential role in seed development. Within the same group, *TfALOG2* from *T. fournieri* showed high expression in leaves. Ectopic expression of *TfALOG2* results in abnormal leaf development, where mesophyll cells fail to form properly at the leaf margins, indicating that *TfALOG2* regulates leaf morphogenesis, which is consistent with its strong expression in leaf tissue. Moreover, *35S:TfALOG2* transgenic plants exhibited no floral phenotype compared to the wild-type, suggesting that *TfALOG2* may specifically regulate leaf development (Xiao et al., 2018).

In Clade V, *RcLSH7* (G6) was highly expressed in the roots, stems, and prickles. Similarly, its homologs, *PhLSH7a* and *PhLSH7b*, were predominantly expressed in the seeds of *P. hybrida*. However, only *PhLSH7b*, when ectopically expressed in *Arabidopsis*, strongly suppressed fruit development, implying a functional divergence between the two homologs (Chen et al., 2019). *RcLSH10a* (G7) and *RcLSH10c* (G8) exhibited similar dynamic expression patterns in shoot apices and were also strongly expressed in prickles, stems, and receptacles, suggesting that these genes may have similar biological functions in regulating organ growth and differentiation in these tissues. However, due to the lack of experimentally validated functional evidence from homologous genes, the specific roles of *RcLSH10a* and *RcLSH10c* remain uncertain and require further characterization.

## Data Availability

The datasets presented in this study can be found in online repositories. The names of the repository/repositories and accession number(s) can be found in the article/**Supplementary Material**.
