# Supplementary material for: Genome-wide identification and characterization of ALOG domain genes in Rosa
Source: Front Plant Sci. 2025 Nov 20;16:1690365. doi: 10.3389/fpls.2025.1690365 (PMC12675423; doi:10.3389/fpls.2025.1690365)
Supplement: Supplementary file 1 [file Table1.doc]

| **Additional File 1. Genome-wide information of *ALOG* genes in four *Rosa* species.** It includes the gene name, genomic DNA sequence ID, chromosomal location, exon region (bp), gene length (bp), ORF length (bp), and protein length (aa). | | | | | | |
| --- | --- | --- | --- | --- | --- | --- |
| **Gene name** | **Sequence ID of genomic DNA** | **Chromosome**  **location** | **Regions of exons (bp)** | **Gene length (bp)** | **ORF length (bp)** | **Protein length (aa)** |
| *RcLSH1* | RcHm_v2.0_Chr3g0449731 | Chr3 | 1437970-1437335(636) | 636 | 636 | 211 |
| *RcLSH2* | RcHm_v2.0_Chr5g0018011 | Chr5 | 12555650-12556393(744) | 744 | 744 | 247 |
| *RcLSH3* | RcHm_v2.0_Chr6g0287901 | Chr6 | 5119860751197972(636) | 636 | 636 | 211 |
| *RcLSH4* | RcHm_v2.0_Chr4g0393491 | Chr4 | 9036363-9036965(603) | 603 | 200 | 200 |
| *RcLSH5* | RcHm_v2.0_Chr5g0070951 | Chr5 | 76771193-76771885(693) | 693 | 693 | 230 |
| *RcLSH7* | RcHm_v2.0_Chr5g0030991 | Chr5 | 24619535-24618954(582) | 582 | 582 | 193 |
| *RcLSH10a* | RcHm_v2.0_Chr2g0129691 | Chr2 | 4551236145512969(609) | 609 | 609 | 202 |
| *RcLSH10c* | RcHm_v2.0_Chr5g0010811 | Chr5 | 7120781-7121338(558) | 558 | 558 | 185 |
| *RrLSH1* | evm.TU.Chr4.199 | Chr4 | 1473204-1472569(636) | 636 | 636 | 211 |
| *RrLSH2* | evm.TU.Chr5.5581 | Chr5 | 62604354-62603611(744) | 744 | 744 | 247 |
| *RrLSH3* | evm.TU.Chr2.2532 | Chr2 | 19622893-19623528(636) | 636 | 636 | 211 |
| *RrLSH4* | evm.TU.Chr1.399 | Chr1 | 4127330-4126728(603) | 603 | 603 | 200 |
| *RrLSH5* | evm.TU.Chr5.982 | Chr5 | 9865814-9865140(675) | 675 | 675 | 224 |
| *RrLSH7* | evm.TU.Chr5.4474 | Chr5 | 51686478-51685897(582) | 582 | 582 | 193 |
| *RrLSH10a* | evm.TU.Chr6.3549 | Chr6 | 34256953-34256345(609) | 609 | 609 | 202 |
| *RrLSH10c* | evm.TU.Chr5.6156 | Chr5 | 67850348-67849791(558) | 558 | 558 | 185 |
| *RmuLSH1* | Rmu_sc0000610.1_g000016.1 | / | 70266-70901(636) | 636 | 636 | 211 |
| *RmuLSH2* | [Rmu_sc0010280.1_g000005.1](https://www.rosaceae.org/jbrowse/index.html?data=data/rosa/rmultiflora_v1.0&loc=Rmu_sc0010280.1_g000005.1:31..197&addFeatures=[{) | / | 31410-32159(750) | 750 | 750 | 249 |
| *RmuLSH3* | [Rmu_sc0003163.1_g000004.1](https://www.rosaceae.org/jbrowse/index.html?data=data/rosa/rmultiflora_v1.0&loc=Rmu_sc0003163.1_g000004.1:37..210&addFeatures=[{) | / | 23130-22495(636) | 636 | 636 | 211 |
| *RmuLSH5* | [Rmu_sc0006203.1_g000005.1](https://www.rosaceae.org/jbrowse/index.html?data=data/rosa/rmultiflora_v1.0&loc=Rmu_sc0006203.1_g000005.1:23..196&addFeatures=[{) | / | 35270-35956(687) | 687 | 687 | 228 |
| *RmuLSH7* | [Rmu_sc0001974.1_g000001.1](https://www.rosaceae.org/jbrowse/index.html?data=data/rosa/rmultiflora_v1.0&loc=Rmu_sc0001974.1_g000001.1:19..185&addFeatures=[{) | / | 3137-3718(582) | 582 | 582 | 193 |
| *RmuLSH10a* | [Rmu_sc0001433.1_g000025.1](https://www.rosaceae.org/jbrowse/index.html?data=data/rosa/rmultiflora_v1.0&loc=Rmu_sc0001433.1_g000025.1:17..183&addFeatures=[{) | / | 133314-133922(609) | 609 | 609 | 202 |
| *RmuLSH10b* | Rmu_sc0003419.1_g000031.1 | / | 103891-103334(558) | 558 | 558 | 185 |
| *RmuLSH10c* | [Rmu_sc0000109.1_g000015.1](https://www.rosaceae.org/jbrowse/index.html?data=data/rosa/rmultiflora_v1.0&loc=Rmu_sc0000109.1_g000015.1:13..187&addFeatures=[{) | / | 60670-60113(558) | 558 | 558 | 185 |
| *RwLSH1* | Rw3G001750.1 | Chr3 | 1489762-1489127(636) | 636 | 636 | 211 |
| *RwLSH2* | Rw5G011540.1 | Chr5 | 13298455-13299204(750) | 750 | 750 | 249 |
| *RwLSH3* | Rw6G026140.1 | Chr6 | 49533631-49532996(636) | 636 | 636 | 211 |
| *RwLSH4* | Rw4G003760.1 | Chr4 | 7297561-7298163(603) | 603 | 603 | 200 |
| *RwLSH5* | Rw5G043250.1 | Chr5 | 75759573-75760259(687) | 687 | 687 | 228 |
| *RwLSH7* | Rw5G019700.1 | Chr5 | 25641067-25640483(585) | 585 | 585 | 194 |
| *RwLSH10a* | Rw2G027830 | Chr2 | 44100062-44099454(609) | 609 | 609 | 202 |
| *RwLSH10b* | Rw2G013630.1 | Chr2 | 14755450-14756007(558) | 558 | 558 | 185 |
| *RwLSH10c* | Rw0G022240.1 | / | 61093-60536(558) | 558 | 558 | 185 |
|  | Rw0G017960.1 | / | 255357-254800(558) | 558 | 558 | 185 |
