# Supplementary material for: Genome-wide identification and characterization of ALOG domain genes in Rosa
Source: Front Plant Sci. 2025 Nov 20;16:1690365. doi: 10.3389/fpls.2025.1690365 (PMC12675423; doi:10.3389/fpls.2025.1690365)
Supplement: Supplementary file 2 [file Table2.doc]

**Additional File 2. Phylogenetic clustering of *ALOG* genes from *Rosa* species and *A. thaliana*.**
A phylogenetic tree was constructed based on the conserved ALOG domains of *Rosa* species and *A. thaliana* using the neighbor-joining method with 1,000 bootstrap replicates. The nomenclature of the *Rosa* ALOG proteins followed that of the closest *Arabidopsis* ALOG homolog, with the corresponding genomic sequence IDs provided in parentheses. Red diamonds represent *Arabidopsis* ALOG proteins, brown circles represent *R. chinensis* ALOG proteins, green squares represent *R. multiflora* ALOG proteins, purple inverted triangles represent *R. rugosa* ALOG proteins, and blue upright triangles represent *R. wichurana* ALOG proteins.
