# Supplementary material for: Genome-wide identification and characterization of ALOG domain genes in Rosa
Source: Front Plant Sci. 2025 Nov 20;16:1690365. doi: 10.3389/fpls.2025.1690365 (PMC12675423; doi:10.3389/fpls.2025.1690365)
Supplement: Supplementary file 3 [file Table3.doc]

| **Additional File 3. Sequences of primers used for cloning and qRT-PCR of *Rosa* *ALOG* genes.** Nucleotide sequences of gene-specific primers used for amplification and expression analysis. | | |
| --- | --- | --- |
| **a** |  |  |
| **Primers** | **Sequence (5’ to 3’)** | **Description** |
| *RcLSH1(fw)*  *RcLSH1(rev)* | CTCGATTCAGTTCCTGTATCTTCCA  GCTTTTCAGCTACTAGCCTTTCCT | full-length CDS and genomic sequences cloning |
| *RcLSH2(fw)*  *RcLSH2(rev)* | GCTCGGCCCCTACTTTATATATTC  ATTACTAAGCAGCTTAGCAGTTCAA | full-length CDS and genomic sequences cloning |
| *RcLSH3(fw)*  *RcLSH3(rev)* | CTCTCCCATCAATCTCATCACATC  GTGCTCAGAGTGGCTACCAACAA | full-length CDS and genomic sequences cloning |
| *RcLSH4(fw)*  *RcLSH4(rev)* | AGATAGATCCGATCCAACTCATT  TATGCTAGAGAGAGTAGTGAGGAT | full-length CDS and genomic sequences cloning |
| *RcLSH5(fw)*  *RcLSH5(rev)* | CAAAACAGAATCATTGTGTAGTCCC  GGTCGATCCAATTATCATCAGAGA | full-length CDS and genomic sequences cloning |
| *RcLSH7(fw)*  *RcLSH7(rev)* | CTTTTCTTTTTTTTCTTCTCTCTTC  TAAAGGGAACTTACTTACTCATCTC | full-length CDS and genomic sequences cloning |
| *RcLSH10a(fw)*  *RcLSH10a(rev)* | CCTCCTAGCCTATTCCTTTCACCT  CATTGAGGCAACAACTACTACGAC | full-length CDS and genomic sequences cloning |
| *RcLSH10c(fw)*  *RcLSH10c(rev)* | TTTTCTTTCAACACTGCACTAACCT  CAGAGGAAGAAATACAGATTACGAC | full-length CDS and genomic sequences cloning |

**b**

| **Primers** | **Sequence (5’ to 3’)** | **Description** |
| --- | --- | --- |
| *RcLSH1(fw)*  *RcLSH1(rev)* | GCGAGAGGAGTGAGCTATGACAAG  CCAAAAACCTAAGATTTTCAAGAAC | qRT-PCR |
| *RcLSH2(fw)*  *RcLSH2(rev)* | CATTTCCTTCTCCGAGATATTTCAT  TGGTTCTTCAGATACTGACCAAAAG | qRT-PCR |
| *RcLSH3(fw)*  *RcLSH3(rev)* | CTCTAACAATGACCCATTGATCT  CAACATAGTTTCAACTTAAAAGCAT | qRT-PCR |
| *RcLSH4(fw)*  *RcLSH4(rev)* | TCAAACTAAATCCTCACTACTCTCT  CTCTTTCATACGTTTCTGTCACTCC | qRT-PCR |
| *RcLSH5(fw)*  *RcLSH5(rev)* | CGGTGCTCCAACTGGTCAATCT  TCAGAGAATCGCCTTGATATACATA | qRT-PCR |
| *RcLSH7(fw)*  *RcLSH7(rev)* | TTCTTGATCCGCAGATGTGAAAATG  ATCTCCAGTGCAGCTTCATCTTTAC | qRT-PCR |
| *RcLSH10a(fw)*  *RcLSH10a(rev)* | AAGGACCTGGAAATAGTGATCGTG  TGACCAAATAGCTAGAGATCAACTT | qRT-PCR |
| *RcLSH10c(fw)*  *RcLSH10c(rev)* | AAGAAGAGGAACAATCAGCTCAAGG  ATATATCCATCTCATGCATCCAAAT | qRT-PCR |
| *RcEF1a(fw)*  *RcEF1a(rev)* | GCCAGAGATTGCCCATATGTA  TCACAGAGTCCTAGCAGCACA | qRT-PCR |
