# Supplementary material for: Genome-wide identification and characterization of ALOG domain genes in Rosa
Source: Front Plant Sci. 2025 Nov 20;16:1690365. doi: 10.3389/fpls.2025.1690365 (PMC12675423; doi:10.3389/fpls.2025.1690365)
Supplement: Supplementary file 4 [file Table4.docx]

| **Additional File 4. Gene information for ALOG proteins used in phylogenetic analysis of nine representative species.** Gene names, sequence IDs, and species of origin. It serves as reference data for constructing a phylogenetic tree, enabling evolutionary comparisons and functional inferences across diverse taxa. | | |
| --- | --- | --- |
| [Abbreviation](C:/Program%20Files%20(x86)/Youdao/Dict/8.4.0.0/resultui/html/index.html#/javascript:;) | Species and gene downloaded website | Sequence ID |
| *AtLSH1* | *Arabidopsis thaliana*  https://www.arabidopsis.org/ | AT5G28490 |
| *AtLSH2* |  | AT3G04510 |
| *AtLSH3* |  | AT2G31160 |
| *AtLSH4* |  | [AT3G23290](https://www.arabidopsis.org/locus?key=36962) |
| *AtLSH5* |  | AT5G58500 |
| *AtLSH6* |  | AT1G07090 |
| *AtLSH7* |  | AT1G78815 |
| *AtLSH8* |  | AT1G16910 |
| *AtLSH9* |  | AT4G18610 |
| *AtLSH10* |  | AT2G42610 |
| *PhLSH1* | *Petunia hybrida*  https://www.ncbi.nlm.nih.gov/gene/ | MK179267 |
| *PhLSH2* |  | MK179268 |
| *PhLSH3a* |  | MK179269 |
| *PhLSH3b* |  | MK179270 |
| *PhLSH4* |  | MK179271 |
| *PhLSH5* |  | MK179272 |
| *PhLSH7a* |  | MK179273 |
| *PhLSH7b* |  | MK179274 |
| *PhLSH10a* |  | MK179275 |
| *PhLSH10b* |  | MK179276 |
| *PhLSH10c* |  | MK179277 |
| *OsG1* | *Oryza sativa*  http://rice.plantbiology.msu.edu | LOC_Os07g04670 |
| *OsG1L1* |  | LOC_Os02g07030 |
| *OsG1L2* |  | LOC_Os06g46030 |
| *OsG1L3* |  | LOC_Os02g41460 |
| *OsG1L4* |  | LOC_Os04g43580 |
| *OsG1L5* |  | LOC_Os10g33780 |
| *OsG1L6* |  | LOC_Os02g56610 |
| *OsG1L7* |  | LOC_Os01g61310 |
| *OsG1L8* |  | LOC_Os05g39500 |
| *OsG1L9* |  | LOC_Os05g28040 |
| *PpTAW1* | *Physcomitrium patens*  https://phytozome.jgi.doe.gov/pz/portal.html | Pp3c8_6310 |
| *PpTAW2* |  | Pp3c23_9660 |
| *PpTAW3* |  | Pp3c20_17990 |
| *PpTAW4* |  | Pp3c24_8490 |
| *FvesLSH1* | *Fragaria vesca*  https://www.rosaceae.org/ | FvesChr6G00002340.1 |
| *FvesLSH2* |  | FvesChr3G00080730.1 |
| *FvesLSH3* |  | FvesChr2G00205870.1 |
| *FvesLSH4* |  | FvesChr4G00131890.1 |
| *FvesLSH7* |  | FvesChr3G00089030.1 |
| *FvesLSH10a* |  | FvesChr6G00034810.1 |
| *FvesLSH10c* |  | FvesChr3G00077380.1 |
| *TfALOG1* | *Torenia fournieri*  https://www.ncbi.nlm.nih.gov/gene/ | MG797507 |
| *TfALOG2* |  | MG797508 |
| *TfALOG3* |  | MG797509 |
| *TfALOG4* |  | MG797510 |
| *TfALOG5* |  | MG797511 |
| *TfALOG6* |  | MG797512 |
| *TfALOG7* |  | MG797513 |
| *LjALOG1* | *Lotus japonicus*  https://www.kazusa.or.jp/lotus/ | Lj5g3v1083950 |
| *LjALOG2* |  | Lj1g3v4515410 |
| *LjALOG3* |  | Lj6g3v0028760 |
| *LjALOG4* |  | Lj1g3v5000590 |
| *LjALOG5* |  | Lj5g3v1353010 |
| *LjALOG6* |  | Lj2g3v2326330 |
| *LjALOG7* |  | Lj0g3v0185409 |
| *LjALOG8* |  | Lj4g3v2604650 |
| *LjALOG9* |  | Lj0g3v0229079 |
| *LjALOG10* |  | Lj3g3v2850080 |
| *LjALOG11* |  | Lj1g3v2612690 |
| *LjALOG12* |  | Lj3g3v1063630 |
| *SolyLSH1a*（*TFAM2*） | *Solanum lycopersicum*  https://solgenomics.sgn.cornell.edu/ | Solyc05g055020 |
| *SolyLSH1b* |  | Solyc04g009980 |
| *SolyLSH2*（*TFAM1*） |  | Solyc02g069510 |
| *SolyLSH3a*（*TFAM11*） |  | Solyc06g083860 |
| *SolyLSH3b*（*TFAM3*） |  | Solyc09g025280 |
| *TMF* |  | Solyc09g090180 |
| *SolyLSH5* |  | Solyc06g082210 |
| *SolyLSH7a* |  | Solyc10g007310 |
| *SolyLSH7b* |  | Solyc07g062470 |
| *SolyLSH7c* |  | Solyc12g014260 |
| *SolyLSH10a* |  | Solyc10g008000 |
| *SolyLSH10c* |  | Solyc02g076820 |
