# Supplementary material for: Genome-wide identification and characterization of ALOG domain genes in Rosa
Source: Front Plant Sci. 2025 Nov 20;16:1690365. doi: 10.3389/fpls.2025.1690365 (PMC12675423; doi:10.3389/fpls.2025.1690365)

**Additional File 5. Amino acid sequences of predicted motifs in *Rosa* ALOG proteins.**
The amino acid sequences of all 20 predicted motifs served as references for structural and functional analyses of ALOG proteins.

Motif1
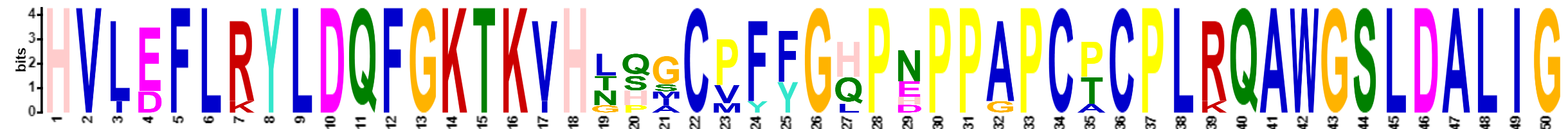


Motif2
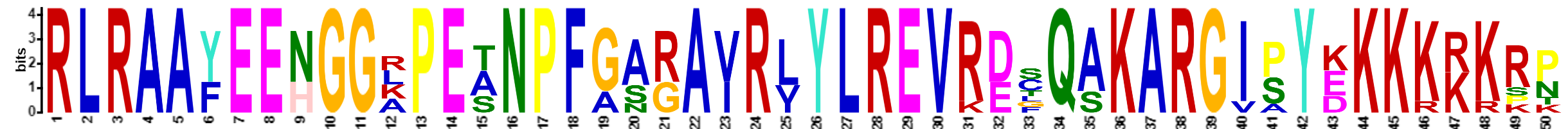


Motif3


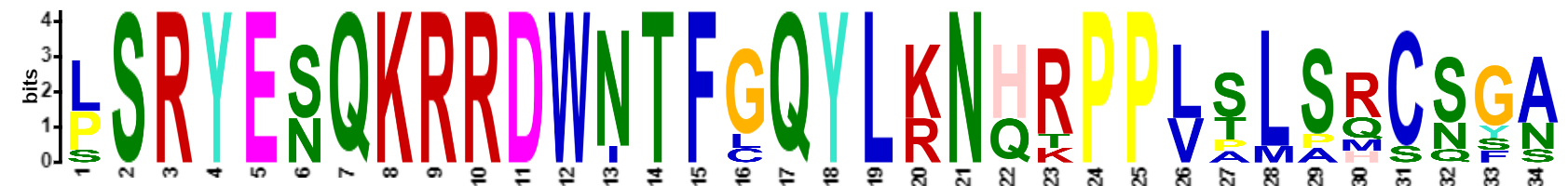


Motif4


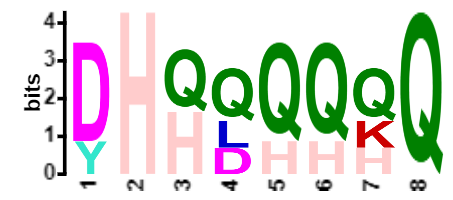


Motif5


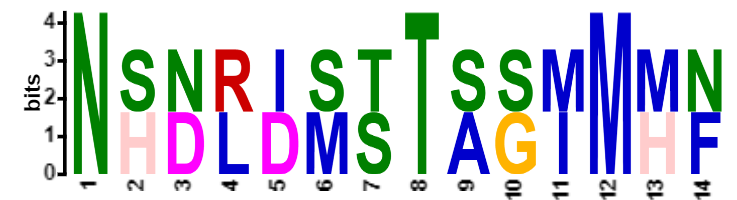


Motif6


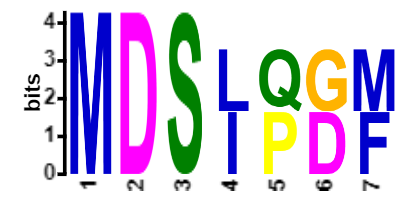


Motif7


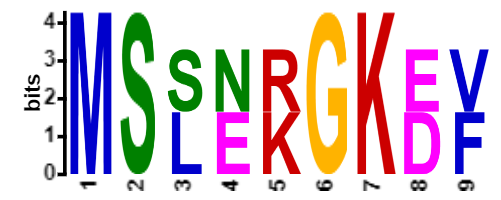


Motif8


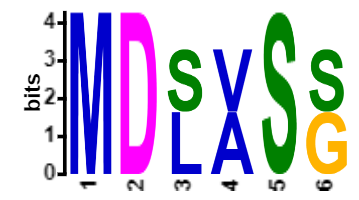


Motif9


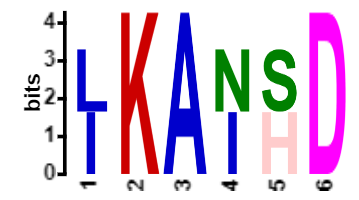


Motif10


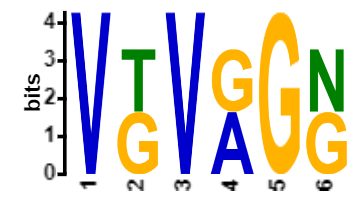


Motif11


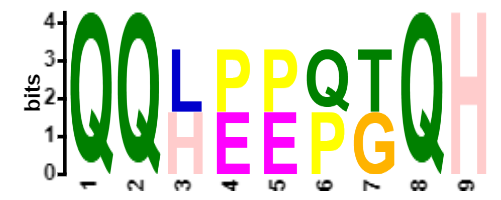


Motif12


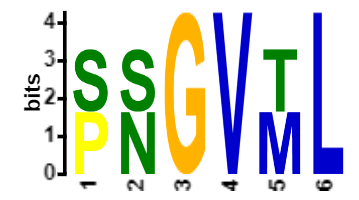


Motif13


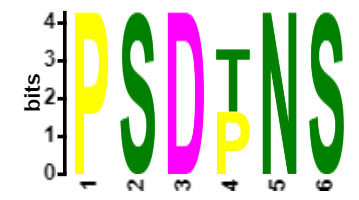


Motif14


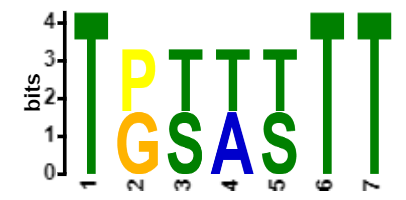


Motif15


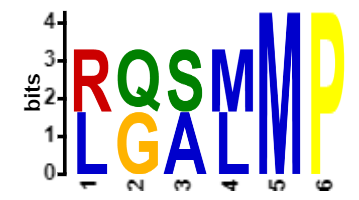


Motif16


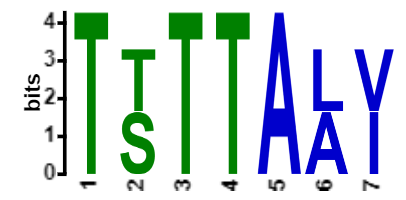


Motif17


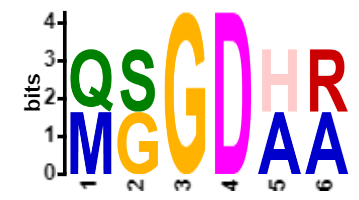


Motif18


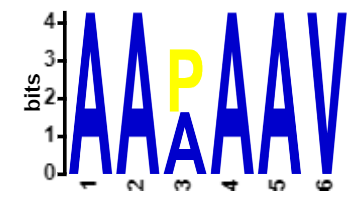


Motif19


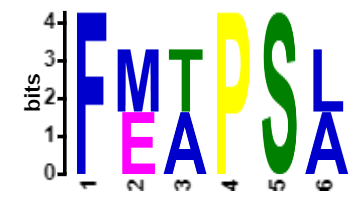


Motif20


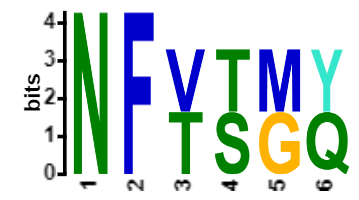

Supplement: Supplementary file 5 [file Table5.doc]
