# Supplementary material for: Genome-wide identification and characterization of ALOG domain genes in Rosa
Source: Front Plant Sci. 2025 Nov 20;16:1690365. doi: 10.3389/fpls.2025.1690365 (PMC12675423; doi:10.3389/fpls.2025.1690365)
Supplement: Supplementary file 6 [file Table6.docx]

**Additional File 6. 2,000 bp promoter sequences of *Rosa* *ALOG* genes.**
Provides the 2,000 bp upstream sequences of *ALOG* genes, serving as a reference for cis-regulatory element analysis and investigation of potential transcriptional regulation.

>RcLSH1 promoter

Ggaaaatgaaagagagcggctagtggtgttttcatcatcacacttgggatttggaggacaaatggctcgttgaactgaaacaaggaccaacaaggcaacaatggggtgtagtacagtggaaagcgatggtttagttgtagtgactaaaatgccaccctagctgagataatagtcataatacttgaaagatgtaggctaggaataggataattcacaaaatgacaggttgcagacttgcagtatatgacactgtttcgagagtactgtaaagtttaacaagcacatccttttcatgcctgtaagggagtggtaaacctgattgtttagggggcatctcatattcattgaaatttgaaaacatatagatcatgatcatggggcaactcatactagtcaaggatcataatgagttaactacctacataaagagtgcattttgccacagcaatccaaatcaagaaatagaggatggtacaaaatttgggatgagatcgtaaaataggtaattgctttgaagtttcctgcatttgtttcggtacatcatattatgttgctaattaattaacaagctatcaaatgcagtttctcctggtttgctattgtgaatagatttccatacctcagaagtaaccacctttaacacccaaattaaggtttgctcatgctctatctcttaattaatgataatagtgagtctgtcaggtgtatcatcattatcactagctacttacgtgccacataatacttcatagttgtacaatactaccatcaccaaggaggaaatggacatgggtttgaagaatttttttttcaaaagtgatataatatatcaattttttatttaattttattaatgacaaattatctaatcaaacgatgtagtgttttattaacgatgtaatatttaataaatattaaaattatgtgaaaaaatgaccatacatttctttttatgaatagagttttttttaaagaaaaataaatatgcattattttattgacaggttgtggatttctaaccatttactaacttgaaaagatgatctcgcataatcatttatttcatattattttttatttattaatcaaacaaagaatagatgcgtaaaaatatctttagaaattggggtttgtttgcacattaattgagacgatttattgaggtgcataaagtattacgtagaaaaaatacggtaaaatttaaaagtaattaggtattatttaaaagttaaaactcaaaactcaaaactaacgctaataataataattaggttgtgtgtttcactcgctttggagaggagttggtggttttatttatatcaatgttatgtatgtcagtgtctgtgtggcaatggtcaccatcaactctgcccactgcctaacttgtcctattaaggcaaaatacttttcctccgactttacttcttcctcattaactttatttcaactcccagtccctcttaacacagactgaacagacatctcccttccctcccttcactccctctctcttatccctctctctctctttctctcacagccctatgtcaaataactctctcagagtctcaggtgcttccctttctcctcaaaggtaaaatgatgcatcatcatataatgatctgtgaactgagacttccctccctcagaaatcaatttccgaactgcagtcttcccaagatttcctgcaggctaattagtcactcgaaccagcttattaagttggctagtttcctcaactagattcccaaacctcgattcagttcctgtatcttccatctctgacaattttatttatggaaaccatgcaccgtcgcaccttatcttcttttggccgatgttaaactccactcctacaagcataagattttctctctcaagtcatatcactcactttagagacaacaccgcccagaaagagaaaaccctaggaattaaaaccaaggagaagaagaagaagacccttttctccaacctcaacatcaacaccaacaccagcc

>RcLSH2 promoter

tactgtgcactatttactatttactgttcactgttccggcttttgaaatagaagcaaaaccctagaccaaaataagggatttgcagctgagatcttggtgacccacgtgaaaaagtccagtgctgggtagccaaatctggcaagcaacaatgaaaagtgggactacggttagagtcttttctgatctgtgattgattcattcaatcggaaaaaccagaaccagtggacagaaagaaaataagagagggagaaaagaaaaagaggacgaaaaatcaagtaccctagtctcatcggatttgacaggtttgacaagatggaacattgcccccaccttctcctcctcaactctattctacccaactcattcattttcagtgctctacaataaataagatgcaaagcaggaagcaacaaaagtctttgggcaggggttgccactgcactgtcaatcaatatgagtgatccgtgtactctaatcatgttggggttttgattacctttacctccacaaatgcttcttttgaggattctctacgtgcccatttttttccttctaccctttctttactttgttctagctaaaaggtacaaaatctttctactgtggagtgattcataatgttacttatttgataataaaagtcgtaaaagttgataattccactcgaatgatgagcaccatattattgatagtgtttcaaaatcaaaattccatcaagtgatggcgaaataaaagagattttaacaatacgcaactgtaaagttataactttatgatatcattttattttaaaaatcttgtaagatcatgtaaacctacatataatggtcaatgttgtctcttaaaataatgatataccgatacaaatacttaaatatacaaaaagtttaaaatatataaatttggtagctcaaataatcaaaaacctagaaaaacatgcacataagattaacgggaacacatttaagatttaaaccacaatagaaactcttcaccatttttttttgcacaattttgcttagtaaaccttaaacaattgaacatttttttaatacaagaaggttccatactaagctgtaaaaactattaatgttggctacttataagaaataatctaccgatttaaacacctagatatacaaaaatcatacgtttaaaattattataacttgaaattttcactttaaaagacaattttacggatacatataactcaaattactgacttcacaggacttgggcatgcccctgagtaacatttggaatcatcatcttaccctgtacgtatattcatctagcatcattaacttcggctgtattcgtttaattggttctttataaacatatatatccacactctgctaaactgtttcttgactcctatctcatctgcctaataaaacatctgcctaataaaaccttaaacagtcaaaactcttttctttggaacaaagaatgtactgtgcaatagtctagggaggtggagaaggccgaaaagcttcaaattgccacaccaaaatggcaaaattaggtcacaaaagtctacaccaccaagtcacgtccagcgtaatcagacttacagatattatgatcaagtttcctctagtgcttactattcacaaaagaagcaaaaattaaaggtcctggtttcgtacatgagagatttgacgttttgggaagtgatgagattcaaactgtcatcaccttgcgtcgggccctcccttcccctgtctccttccttcatggccctctctaccGTCCTCCTTCATTCCTCTCTCTCTGAAATAATGTCTCCTTTTCTCCTTGCTCGGCCCCTACTTTATATATTCTTACACGTCTATATATCTCTCCTCCATCAGTAACTCATCGTTCTCTTCATATCTGGCTAGCTAATCCAAAACCCCAATCTCCAAAATCAAAGCCATTTCCTTCTCCGAGATATTTCATCCAACACTTGGTATAACACCGGCGATCCTACATACCTAGTTTTTCTTTTAAC

>RcLSH3 promoter

agaggggctggttgtgtagggttttccattctaggttgtagcctttggcatgtgtggaagaggaacatgtttggttggtcatagcttcttcttttttttctttttataatttttttattgggatcacacagtattctctaaggctttctaggtctaaagactaattcgtgcccagagatcatgttagaatgttttcttcccactggccaccaagaatagattggttttaacttcaatcagcgtcggcaggattcaaacccgagtgtgggggtaccacacctgaaggctcttaccaactcaaccacatgtgacggtttggttttttgtagctagcttctcttgatgtcattctatcatatgaagtttcttcaaaacttaatgccaacttcagtctccgaaactatgagatcccttaaaatatcatgcttactgtttttatagaaaagagaatgtatcactttttcatattttctcattatcttaaagaaaatgtgtaaatatagaaatgattcatgataatttaaacctgtactctttttacttctttttttgtttttaaccacttctctcgtctagcttatcttcagttcttctttaattgacaacaaataaaattatatggaaaagtataaatacgagcgatcaattatatgaaagcatttacaatcgtaggactgagaggcaacctaagattaaaagtaaatcaagcggctggaaatgtgtttaacactgttgtccgtatatctcacccaatatatccaaacctctaaaattttcttgacattggggcattctgcaaaaaatcataagtcccacagggattccatatatgtataggtgactgttccaagtgtttaaaagctgagatggggccaaatttgtgtgtcaggaattgaagtcttattttggggagaaaaagaaaagaagtactgggagacaaaaaattcacaagggcactctagtcattcagaaaggaactgtcattcaattcaacagtagagcttcgagggtactagtcaagaaagataatcgtgttatataatctataatccctttagtcttgcgtggggcagctgtaccacgtggccgtgcagttgtccaatcagtattcagtagggggtattttagatattttatttttaaaaagtaggggtagtttcaaaataaggtaaaaaatttatggtgtgtgattggtgaatcgtaccacgtgtctggtatgcataacgtactcaagaatttttcatccctttaaatgttttaccactatccacacttgtATTTCCTGTCTCTATTTCAACCCACTGTCCCAAATCCCTTTCCACTTTCACAAAAAGCCCTAAATGCACAATTCGATCCCTTCTCTGCCACAGTGTCTCTCTTCCCTAAGAAGAGGAACAATAATTGTAATCCATTATTAAGAGTTGTGTTTGCTCAACCCTTTTCAGGAAAACCAGCCCCTTCGCATGTTAAGAAACCAAACCCAGCTGCCTGGAATTCCATCAGAAAATCCAGATCCTTATTCAAGCTTTCTCTTTAGCTAGTAGTATCACTGTTGATTCTCAAACAATTTAAGCAAACAAAAAAAAAGTACTTGAAAACTCTGTGTGTGTGTGTGTCTCTCTCTCTCTCTCTGAGAGAGAAAGAGCCAAGAGGTGCAGTCCTCTCAGAGCTCTCCCATCAATCTCATCACATCCACAACAAAAATCATCATAATCTTTTTCTGCTTCACCATTTCCAGCTCTATTTCCTTTTCTCTCTCTTTCTCTCTCTTTTCACTCCATCCATCTCTTTTCAGAATATGGAATTCCGTCTGTAATATCAATCAATAATTAGGGTCAGTTTTGTTTCCTTGTTTTCATAATCAACACTAGAAATCAATCAAAGATTTATCGAGTACTGTGCCAGTACCACTTCTTCAAAAATTATTTAAACTCAACTCTCTCTTTCTTTGTGGTGAGAATCGGAAACCCTAGAAGAAGAAGAAAGTAATTAGTAAGTCC

>RcLSH4 promoter

cttataaaatataaaataaaacccagacacacctttttaagtccatggaattaaaaccctagaagagtttttctctcaacgtcgccctagcctccacatcctagcttccttctatcactctcttctcaacttctctccacccaatggcctccattgatgctgtcaccgcaagcttcgctacttcccttgcactcgctgaatgaggcagcgctcctgacctagggaagattggtgatggtcttgttctcaggtcctcccaatccttcctgattggaaaacctctgacccgcaagccggttgatcctacggcattcaaagttcactttcaccgcacttggatggtaggaagaccacttgggagcaagaacaagaatccttgctcacaaaagaaggtgggtatggctccattgcgtctcacttaccctactactgctgttgttcaggaggttagccctaagggcaaagggaagctctaagctttctagttctcgatacctagcatttgaattctagtcttttgctatttaagtttaacttctctaattgtacactaattgaggtctttaggcgattagggttgctctagcaaggatttagttagtgttggatttgtatatggctaggtctaaaaattgtgctcattttgttgacaatgagggttactagtcatttgcttggtagcttaggtcatctaggttttatttggtgtaagacaacatataatgtatgtggcttctggccatggataagtaatgaaattatctattcctaaaaaaaaaacaaaacaaaacaaaatggaacacacctttgccgatgagttgttctccgacaaactttcctaatatataaatcgtcaacaaaaattatttataaaaaataaaagccaatagattaaaagtttgaaacctctgcagatcaatcatccgttagtaaagaccacccataaaaaaattattaaaaaaaaaaaactaaaaaatcagacatattacgataaataaattatcaacaaaaaatcacccataaaattattaaaaagtatacacattatccgtcggaaaaggcttcacatttgcctctctaacaaatgtcatatgcatgttaattgttaacatgctcataagtatatgcaccatgtctaactggctaaatctgttcgttgtataacaccaatcatataccagatatttgtcaagagaataatgatctacgactgaagaactgaagatgatactcatgatagtaatacaaatggttggcagttggcacatcatacgggccttgacttgaaattgtgtaattatatgcaacttgatactgattactgaataatgaaaattcatttgccaaaaaaataagagaataatgaaactgaatggagggtaaagtagtcattaacaaaaaggtgttgtcaatcaatacaaaagggtaaagaaacaatatcatgttattcccttcacaTTTTTTTAACCCAAACACCTTTATCCTGTCTCCATCCCATCATCTCTCTCTCTCCACCTCACTGTCAACCTCTTTCCACTTACACAGAAACCCTACATGACTTGATACATCTTTTCCCACTTACCAAATTTATTAATTTACCATCAAATCTCTCTCTCTCTCCCTCCCTCCCTCTCTCTATCTTTTCTGAGGTTGTAGGGCTATGAAGAACTGAGTTTCTTGCCCTTTTCTCGGAAAAAAAAATCTGGCAATCTCTCTCGAAATGTTCCCATGAAACAAAGTTAGCAAAAGCTAGCCATACCCCCACATCATAGATTTCTAGGTTTGATCATACAAAGATTCAAAGAAAACCTAGGAAGGAAGGGCAAGAGAGATTCATAACCCTACTGTTCTTTTCCCACTCCTATATGTCAAACCACATCATCACCTTCATGAAAAACCCAGTTCAAATTAGATAGATCCGATCCAACTCATTTTGATGTCCTACTTCTAAGTTCATCACCTTCTCTCTTTTTGATTTTGAGTTTTGAGTGCTGGAATCC

>RcLSH5 promoter

tatcattttataaattggtatgacgtttctaaccatacattttaaaaggtcattgtttatgtgtaatcttgtattagtttatacatacgtactatttgataggttcttcgaacttataaaacaaaaaaaaatgtattttttagttaataaataagtacacaaaacttgtgcaattaaaaatacatacatatataatacattagagtcatagatagtttgtaaaaagttagcaatataataagtatgataattgtcaatttggcacatctcaactattgattcactccttcctccctcaccgtcacgaatgcttaacggctttgtcaaacttatgaaaggtaaatcattagtgtcaagaatttttcaagtgagcacataggaaaaattaagaatcaaaccataccacctataaagaaatacaaaaaggatgttgttgacgtttttagtgaggcttcatgtagtagtacgaataaaaaaacatcattatgatagttttatctacaaaattgcaagtatgataaaaacagtgaattagtgcaaaaaaaatcattaacggtgcgacgaataacgttgtccaaattttactcttagggcaactccaaccataggctctatttgggggtgctattctcattttagcacccccttgttgcattattcatgtatgacttaattctcatcttcaacaatgaggtgctatatgggtgctattttcactattcttgattaaatataatatgagtgtaatgttgatgaatattatattaaaagtatgttaatatagttaaataaggtaaacaattgtgaaggagatggaggatatttggtgtgaggaaatagaaaagaatgggttgatatttatagaatttcccacaatttactgttccaataggtatatatttttttttccctcaattttctggtaattttaaaaaaaatttggaacaaaatagaaataataacccttgatttaataagagtcattgatcactttttttccttaaatctgagccgtcagattaaataaggtcaatttgagtaaaaacaaataataacccatttgctgggccctatgaacagtatccgagtcttggtcttggaaaagtctcaaatatgagacttgggatgagcccaagtcctataattttaggaccaactccccctaaaaaatattgttggaaatgaaaagtcttataattcaacaaaatctagttttaaatcctatggttggagttgcccttaatgcccgttaactattaatcttagctccagaattaagctgcatgaacaaagataataattaattttttcttttgtgcggtaaaaaaaaaagaaagtaaaagttaaaatgaggtgtagtttagtgaataagcgccctgtggtgagtataaatggaaagtggtaaaaaaagggtaatggattgctactgcagatagggattagggagttgaatagtaaatatgtcacttccattggggccaccatgtgaaaatatttgtgaataccaccttccttcttcagctccttctatctatacccctcgatctcactacacaaaCCCTCCCCTTCACTCCCTCTCTCTCTCTCTTTCGTTTTCTTAGATGAGAGTGAGAGTCTCATTCATCATATTCATTCATCCATACTTTTCACGTCCCATCACCAAACGCTCCCCTCTTTCTTCCCTCTCTCTCTCTCTCTCTCTCTCTCTCTCTCTCTCTAAAACCCATTTCTTAGCTTTCTCTCTCTAAAACCCTCTATTTCTCAAACACAAAACAAAAACAAAAACAAAAAACTCAAAGAACTTTTCCCGTGAAAAATCCACAGTAACTCACCCCTCCCCCCTGAAGCTCCCAATTCTCATTTCACATGGAGGAAGGGAGAGAGAGCTAGCTACACATATATAAATATATAGCTTATATTCTTAGAGAGCTAGCTAGCCCACAGATACATACCCAATTGCTAAACAAAACAGAATCATTGTGTAGTCCCTGTTCTTCTTG

>RcLSH7 promoter

gattgtagcaatcggcatccaacttagaaattccacgtaattttcaagtaaatttttggaagttttacgtgttttcttcatttttgaattttttgacatgaattgtcaaaaacaactgtcggtacccgttgtggctcctaatgctcattatcaattgttagaatgtctacccacataacaacaaattaaaaccctgttcttcttatcaatcaaattataaggaaattttttttttcaatttcaatcggttttctattgactaattctagtgtaccaaacaaaattgtttgatcattgggccttgcttatgtcttactcaaagaattgaaagggaatttaacatgttgctagaattttggaaacagacctttaaggtattgcatttggcccgtatcctagtatgtgataaagggcaaaaaggataagggaaacaacaaaaacatcaccacaacaaacactagtacattttagatattatttataagtatcatcactaagcattattggcagtggtgatggttttcctatttataagaaagaaatttctttttgtgacaggacctcctccacaaagcaccgaaacctcagcttgatgacacacgtgtaggcgatttaattatatattgttgagcatttagaatacttaagccaagttggtaatgatcattattttgtttaatataacttttgctgtaatggaaaatctttgagggtcaaccagctcatcttaaacactgtatataatctcaccatccacctacaaaaagtcaaaaatcaaactgttcaaactatgttggagactaggtaggacttacaatttaatttgagttattggtctttgtttgtgtagcattcaaaaggaaccaatcccttggaatgaaataatttggatccattacagtaataaatgataacaaaacgtgttggcaagcaattgaacaagatagtccataacaaaacaagatgtatggcaaaactaattttaccctagctattctctcttatgcatctttagttttctcaattgagagtgatgatgtttgtcttttctttaaattttgtaggatgtatatatttatgaaaaaatatttggtgcaccaacgtacacttacaccaacataaatagacggttaggtaacattaagtatatatttttattattaaaaggaatgttgtctataaatatcaactgttgagatttactgtatctgttggttcacttacactgttagtgtatagaagaatttttcatctttcttccaaagacattttgtggcatttttattttcaaaaaaaaaaaaattgtggcattttaatcccacataatcgctttagaattgtgtgttccttctttattctttttttcctatgatcaaaataaaaatactaaggtgattatatggatatatcaaaagcaaataacacgtagatgcttgatgtcgtgaataatagtagtcattagtcattctgtgaggtaccggatagaatccttcattagttattgcataattctattacaaaaataatataattgatcctctaggatacattattagactcaccctagactagaagtgaaatgagtaatctggactctcgagagtaaatgcaaacgctaaacgcctaatcccaatctattaactcgggttacctagtccacaggtaatgaaccatagtactaattacgctaatatagtcgataatgattgctttgatgatggaagcacaataataaagatatggaaaaacaataaactactattaacactatctaatagtaccattaaagcaagaaaataatttgagagaggaagaaaaagaggagctaaagcactttcattggtcccataccatgtctcctcactagccatagccccccccttgaTGTGATCTCCAAATATTTTTTTTTCTTTTCTTTTCTTTTTTTTCTTCTCTCTTCCACTCAAATTTTGTTTTCTTCTCCCCCCAAATTCCTTTGCAGCTTCACTCGCCCAATAATC

>RcLSH10a promoter

tttacacacatactttaagaggaataagtttgttattagccatgcactaaacaagaataatagaagagagttcaatttaaatttgagacgtggcaaagaaagaaagaaagaaaataaagaagaaaaaccgaatgcatgaacatgcccaaaaaagttatgaactaattacagcatgagttctgaacaggctgtaattcattgtttatgaatatatatatatttaataataatgtaggattcctgacttcctgtgctctttctggttttgtttctttgacaaagacatcttaaaggtgagggcacgaacagtactgtatcttaatagaagatctcagtctcatctactcttccaccgaataagtcaccgaataagtcaaattaattttcatagtacataataaagcgagtccccaaatctacatagtacataacaaacggagtccccaaaaccttcatatatacttcaatgcactattatgacaatggttatcaaagaaacaacaagaaaagcagctgcggcactatcatcactattatatatataattatttttcttttttgttttcatatgttattatgaatactttgaagaaaaaggaatctgcactgcatcctacatattaaacaaaaaaagaaagaaaataaaccacatagaaaaggatcaagaagttctctggtgagaacaaggataccctagctagtctagggtttcaaaaagggcttctctgtgtctggcgggtcaatagtttttggtgtcaatcaaaaccctaatcgcagcgggagcatgggttttgttcttgtttggaccacaaaataatataggctcgaataagtatcaacgtagttagtaaatccctaattgtcactagccttgacaaagttgtctcgtaccataattgcaagccttgacaaacatcacgtatggaatatatgggaagtaatgcgaatgcttctattagggactatataatgtttagttaaactgactgcatttatttttaactatatgtatgttctggtgatactctaatgagtatcatgtgttagaaatttactctccgttgggttgttttagaattttttttttcattgtacatgggtctaggttccaatgtatgtgatgatttcgtgttgaccaattggttactaaataatattattctttatgctcaagatagaaaaaaatacgtagaaagaaacgcatgtggtcagaattatatcgtcaaccttcattaagttatctttccaataaaatgaaccgtgtatcagcaaacatgaaacaattttatgcatttaaaacttaatatttattaattttaaaatttaagtacaaatgcaaagaacttacggagaataatcttaccctaaactttaaagaatcaatttcattgtcctataaaaactctaaactctaaacccttcaaattcaatatttgttatcttggccataaaaacttgatgtgattaaggatgtctggcttaaatttgggggatcatgagatgattacaaagtgatgaccaaattcaaggaaaattcttaaggctttaaaaatttataaatgaaacataaggcgcgcgtccatataaaagacaaggacaaatacttttgtatgcttattgagggaaacgtagcgttcatgctaatggcactaatagaaaaaaaaaatagaaaaaaaaaagaaaaaagaagaagaagcagcacatatagaaatgaagaaaaaattgaaaacagaaagcaactcatatgaatattggcccccaTGGCAGGTCTTCTCACTGGGTCCCATAAAACAATGTGAGAAGAAGATCTCCATCTTCTTCCAAATCCCTCAATCCCCCTCTTTTTAATTTTGCCTCCTCCTAGCCTATTCCTTTCACCTCTCTCAGACCCTCCATTATTTGCTAGCTTTGCTTATTTAGCAAAAAAAAAAAAAATCAAGCTAGTTCATCAGGTTCTCCACTGATCCATCAATCCATCATCGACGATCCATCACTGCGAG

>RcLSH10c promoter

gccaatgaaaggaaaattattcccaaaggaaaggaaaaagaggaggaaactggttcctctccacccacctcaggaaactagttttcccccacggccccatccacttgcaaaattatatgactatattgtccctgctcaaaatcattatttgctaatataaccgactgtacttgttatttgtatgatttttacactacccctacaatggaaactaaaatatgtattctgactacattcctagtttttccaaacactggaaacgaaaattgctgggaattgcaatttcccatgcctatggatagagcaaggaattgatttcctttccttttcttttcgcgaaccaaacgcggcctaagtaattgccagctagctcactccacattcactctaattttacaagcctaccctaattcagaaattctggctgctaatcaagttctgtgatataaagtagcctattagaattagggtttggggaaaagagagaagatgtgttctgttactacacgggtttaacaacaaaaggacttctaaattatttaatgaactataccattttggatacatgcatctagtgatactgcactagaaccagaaaagccttgatgttcgatctcttggatttaccataggagattattcagactcgaccatttccaaattgaactcgtaaactttccgagcgtataatgcactttgatcatgaattcttgtcattattcaaacaccacctggcttacattaaaatttacacagcaggatttaaggaatatagagctatattcctatgactttttttgtgaccattcatgtagttcatgaaaaagggcagaggacagcgacatccgcaacgaacattgcccgagtaacaacttaaatacacaaatttcaaggggaaacaaatagtagacacattaaaataataatacaaggatatgtattattggtcgttaaaatttaaaataagataacttagtacgtgggtgaatagatagattctcaaaaccctgatactattgttttttaccataatagaaggctagctttggctcttgtgcttaccaagtgcaactactattagctaagggtggaaatcgattcataatagttttattctcaaaaccccgataccttgtcaatattatgtagagactagagtatcaaattttaaacaataatgggtaaggaagctcaaaatcttgtaaatttataataattatgattaagtttttatgttagataaacggtcacaaatactaacgcgtattctcttactgttctacctaccgcgataaatatcggtccttttttatatcatagttgtggtagactaacttgacacatgaagttatcattgatgtatgtgtaaaactgttctatcatgtgtcattaatgttaatatatcgaagtagctaaaagatccctaattattgatactaagtagttttagcagtgtatctgtcctattgaatgagtaaacttctatttcttttccctgtacatatgaaattaaccagggtccatatatatatatatatatatattatgtcatgatcatcatatattcatattgctgactacggatgaaaatgagtaatcattgcatggatgatagcataggcataacattattctacccatatttttccttggatttgttttggatgatgagtaccataccacacattataattatatattcccaagaaacaagaaacaatataatactactcagtgctaatagtgtcactataaaaatgagagagatccaagtgagaagcggagagggattgagtgtttagagctagatagctaagctaggaatagaaaaagaggaacataaagcattttcacagggcccacgacatgtcctctcaatagacccaaagatgggatttcgatttctatcttttctttcaacactgcactaaCCTATTCTTCCCATACCTCTGTCTTCATTCTTTTTTCACCTCCTACCATTTCTCCAATTCTTCCCAAAC
