# Supplementary material for: Genome-wide identification and characterization of ALOG domain genes in Rosa
Source: Front Plant Sci. 2025 Nov 20;16:1690365. doi: 10.3389/fpls.2025.1690365 (PMC12675423; doi:10.3389/fpls.2025.1690365)

**Additional File 7. Quantitative expression data of *Rosa* *ALOG* genes across tissues and developmental stages.**
The data were used to generate a heat map using TBtools, highlighting tissue- and stage-specific expression patterns.


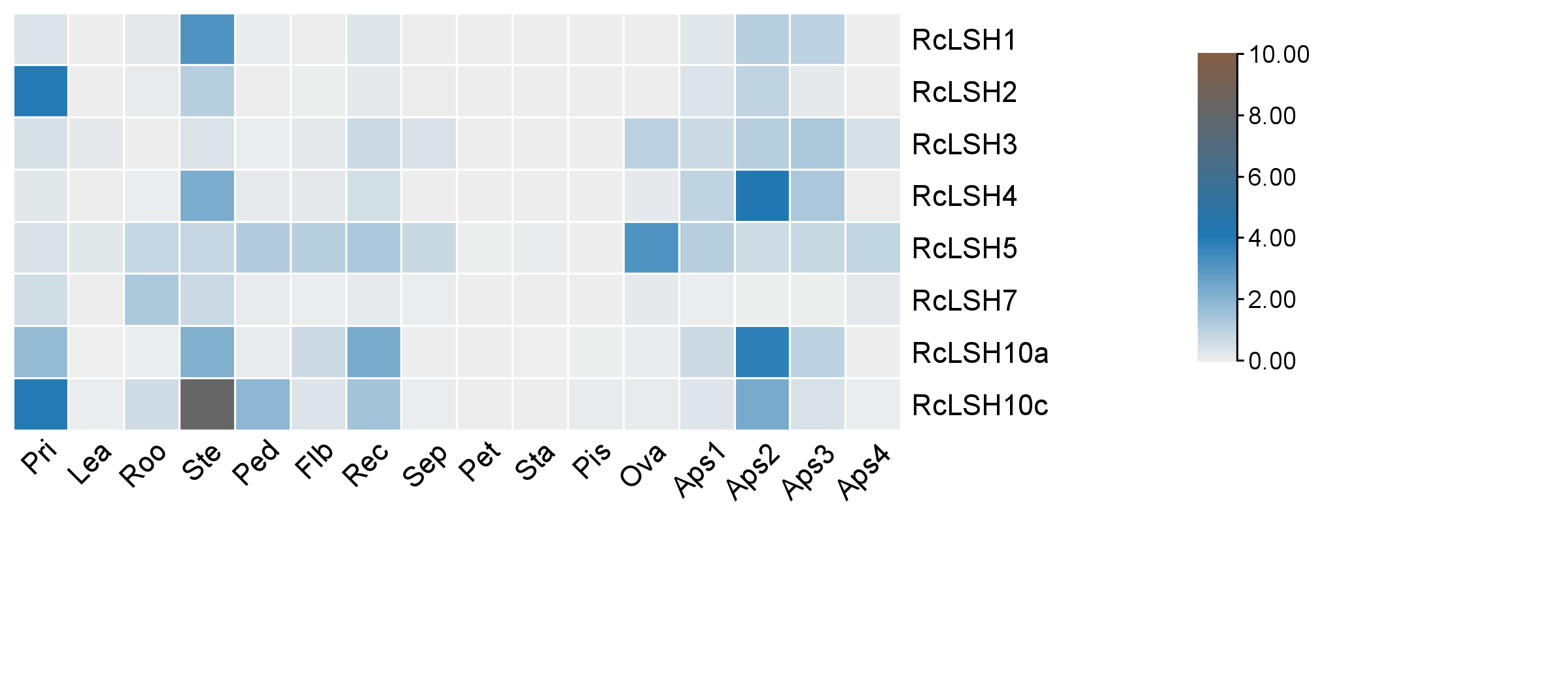

Supplement: Supplementary file 7 [file Table7.doc]
